# Collagen scaffolds derived from bovine skin loaded with MSC optimized M1 macrophages remodeling and chronic diabetic wounds healing

**DOI:** 10.1002/btm2.10467

**Published:** 2022-12-07

**Authors:** Hengdeng Liu, Ronghua Yang, Shixin Zhao, Fei Zhou, Yiling Liu, Ziheng Zhou, Lei Chen, Julin Xie

**Affiliations:** ^1^ Department of Burns, Laboratory of General Surgery The First Affiliated Hospital of Sun Yat‐Sen University Guangzhou Guangdong China; ^2^ Guangdong Provincial Engineering Technology Research Center of Burn and Wound Accurate Diagnosis and Treatment Key Technology and Series of Products Sun Yat‐Sen University Guangzhou Guangdong China; ^3^ Institute of Precision Medicine, The First Affiliated Hospital Sun Yat‐Sen University Guangzhou Guangdong China; ^4^ Department of Burn and Plastic Surgery Guangzhou First People's Hospital, South China University of Technology Guangzhou Guangdong China

**Keywords:** anti‐inflammatory, chronic diabetic wounds, collagen scaffolds, macrophages, recurrence rate

## Abstract

Owing to the persistent inflammatory microenvironment and unsubstantial dermal tissues, chronic diabetic wounds do not heal easily and their recurrence rate is high. Therefore, a dermal substitute that can induce rapid tissue regeneration and inhibit scar formation is urgently required to address this concern. In this study, we established biologically active dermal substitutes (BADS) by combining novel animal tissue‐derived collagen dermal‐replacement scaffolds (CDRS) and bone marrow mesenchymal stem cells (BMSCs) for the healing and recurrence treatments of chronic diabetic wounds. The collagen scaffolds derived from bovine skin (CBS) displayed good physicochemical properties and superior biocompatibility. CBS loaded with BMSCs (CBS‐MCSs) could inhibit M1 macrophage polarization in vitro. Decreased MMP‐9 and increased Col3 at the protein level were detected in CBS‐MSCs‐treated M1 macrophages, which may be attributed to the suppression of the TNF‐α/NF‐κB signaling pathway (downregulating phospho‐IKKα/β/total IKKα/β, phospho‐IκB/total IκB, and phospho‐NFκB/total NFκB) in M1 macrophages. Moreover, CBS‐MSCs could benefit the transformation of M1 (downregulating iNOS) to M2 (upregulating CD206) macrophages. Wound‐healing evaluations demonstrated that CBS‐MSCs regulated the polarization of macrophages and the balance of inflammatory factors (pro‐inflammatory: IL‐1β, TNF‐α, and MMP‐9; anti‐inflammatory: IL‐10 and TGF‐β3) in db/db mice. Furthermore, CBS‐MSCs facilitated the noncontractile and re‐epithelialized processes, granulation tissue regeneration, and neovascularization of chronic diabetic wounds. Thus, CBS‐MSCs have a potential value for clinical application in promoting the healing of chronic diabetic wounds and preventing the recurrence of ulcers.

**Abbreviations**
BADS: biologically active dermal substitutes
CBS: collagen derived from bovine skin
CBS‐M1: CBS‐MSCs‐treated M1 macrophages
CDRS: collagen dermal replacement scaffolds
CM: conditioned media
Col3: type III collagen
CPT: collagen extracted from porcine tendons
CVM: collagen from porcine visceral membrane
dECM: dermal extracellular matrix
DFU: diabetic foot ulcer
GT: granulation tissue
IGNG: interferon gamma
LI‐M1: LPS/IFNG‐ activated M1 macrophages
LPS: lipopolysaccharide
MSCs‐M1: MSCs‐treated M1 macrophages
NES: Normalized Enrichment Score

## 1 INTRODUCTION

According to the International Diabetes Federation (IDF) report, about 537 million people across the world suffered from diabetes in 2021, with an estimated prevalence rate of 10.5%.^1^ By 2045, this number is expected to increase to 783 million.^2^ Diabetic foot ulcers (DFUs) are a major source of suffering with exorbitant medical costs.^3^ Continuous inflammatory stimulation inhibits the healing of DFU.^4^ The recurrence caused by scar formation after wound healing is also extremely common, with a 3‐year recurrence rate of approximately 60% and a 5‐year recurrence rate of >70%.^5^ Therefore, accelerating the repair of DFU and reducing ulcer recurrence remains a challenging issue in the present medical research scenario.

Although large and deep DFUs reaching the muscles and even the bone tissues can be healed through long‐term conservative treatment, they can cause scarring and are prone to recurrence.^6^, ^7^ Even if autogenous tissue reconstruction (such as a local flap transfer) is used to reduce the healing time, there is a risk of insufficient donor skin or flap and long‐term scar contracture.^8^ Recently, collagen dermal‐replacement scaffolds (CDRS) were recommended for full‐thickness defect wound repair due to host‐cell induction to grow into scaffolds and reconstitute substitute tissues with endogenous cells.^9^, ^10^ However, the structural components of CDRS are similar to those of an acellular dermal matrix, which may be insufficient for certain biological regulation functions, including immunomodulatory and active induction of regeneration.^11^ The application of CDRS in chronic diabetic wounds with ischemia infection is extremely limited.^12^ In addition, the study on the interaction between CDRS and cells in vitro and the macro‐regulation mechanism underlying the healing of chronic diabetic wounds remains to be elucidated.

**SCHEME 1:**
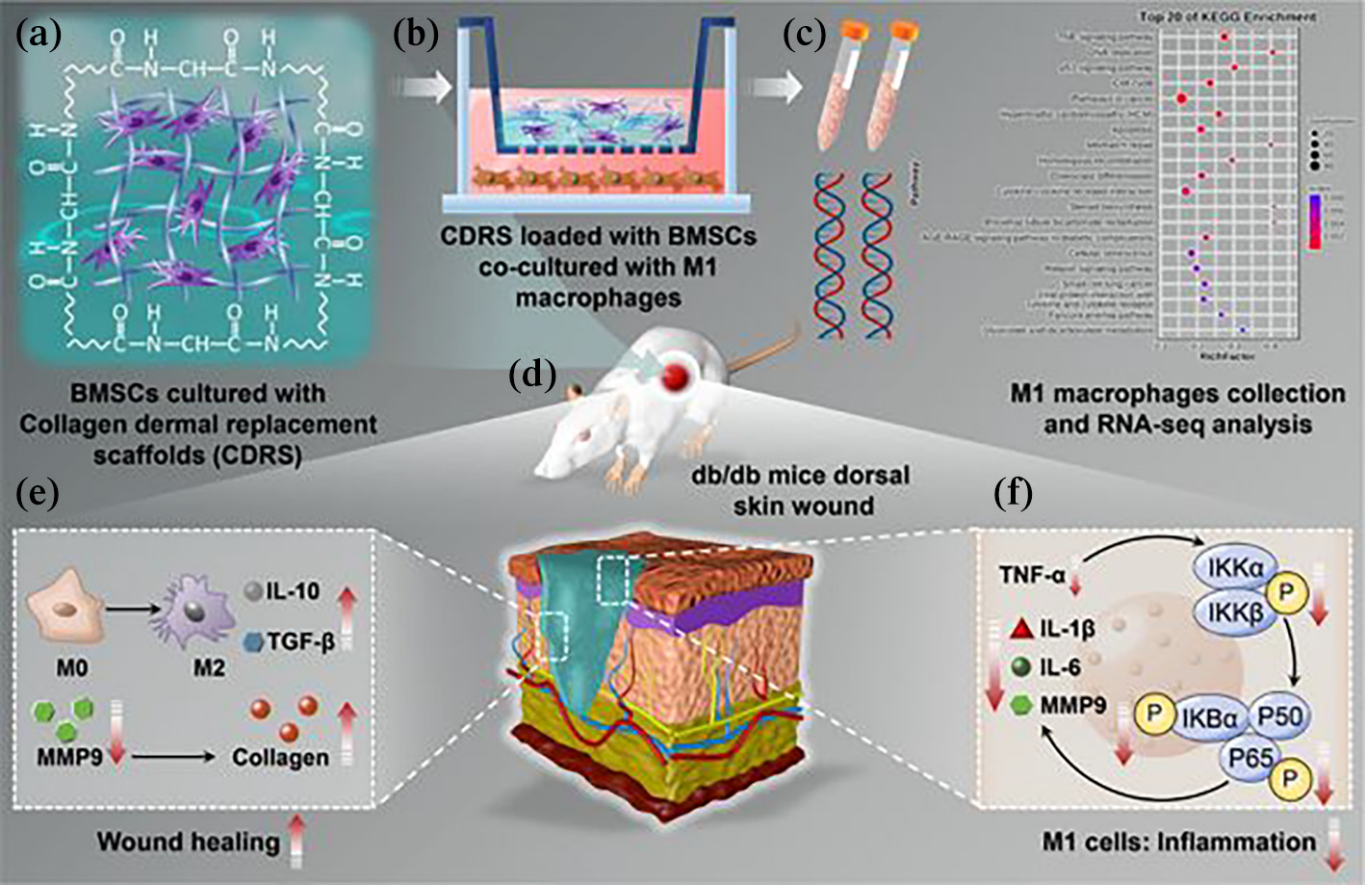
The design pattern of BMSCs implanted into collagen dermal‐replacement scaffolds (CDRS) for repairing chronic diabetic wounds. (a) The formation of biologically active dermal substitutes (BADS). (b) Co‐culture of M1 macrophages with BADS. (c) RNA‐seq analysis and explanation of how BADS regulated M1 macrophages. (d) Establishment for back wound model in db/db mice. (e, f) Modulation for the polarization of macrophages and wound healing in chronic diabetic wounds. BMSC, bone marrow mesenchymal stem cell.

MSCs have emerged as the most valuable multipotent adult stem cells that can be applied in regenerative medicine owing to their multipotential differentiation.^13^, ^14^ Low immunogenicity is helpful for MSCs to participate in tissue regeneration as an allograft.^15^ Moreover, previous studies have reported that the paracrine stocks of MSCs play a major role in skin regeneration, which can mainly be attributed to their involvement in various functions such as immunomodulation, angiogenesis, chemotaxis, and stemness regulation.^16^, ^17^, ^18^ Some promising studies have reported the efficacy and safety of BMSCs‐mediated DFUs therapy, which include several successful clinical trials.^19^, ^20^ CDRS has various properties, such as mechanical stability, biocompatibility, and degradation, which can stimulate cell adhesion and migration as well as promote signal transduction to guide cell activity.^21^ Consequently, we implanted BMSCs into CDRS for establishing biologically active dermal substitutes (BADS) for the healing and recurrence treatment of chronic diabetic wounds (Scheme 1).

The disorder of inflammation in M1 macrophages impairs the function of the reparative cells around the wound, which can be linked to ulcer formation and recurrence.^22^, ^23^ Moreover, the decrease of M2 macrophages reduces the secretion of anti‐inflammatory and growth factors, which further aggravates the difficulty of tissue regeneration.^24^ Therefore, to simulate the continuous inflammatory state of chronic diabetic wounds, LPS/IFNG was used to induce the polarization of M1 macrophages, after which they were co‐cultured with BADS. We confirmed that CBS‐MSCs could inhibit inflammation and promote extracellular matrix (ECM) production by regulating the TNF‐α/NF‐κB/MMP‐9 signal axis in M1 macrophages. Next, we transplanted BADS onto the back wounds of db/db mice and found that CBS‐MSCs achieved noncontractile and re‐epithelialized healing. CBS‐MSCs inhibited the polarization of M1 macrophages and the secretion of pro‐inflammatory factors, as well as promoted the polarization of M2 macrophages and anti‐inflammatory factors. Considering all these factors, our study originally reported that CBS‐MSCs had the potential to reduce ulcer recurrence after the healing of chronic diabetic wounds.

## 2 MATERIALS AND METHODS

The detailed and expanded Section 2 are available in Appendix S1, Supporting Information.

## 3 RESULTS AND DISCUSSION

### 3.1 Characterization of CDRS

**FIGURE 1:**
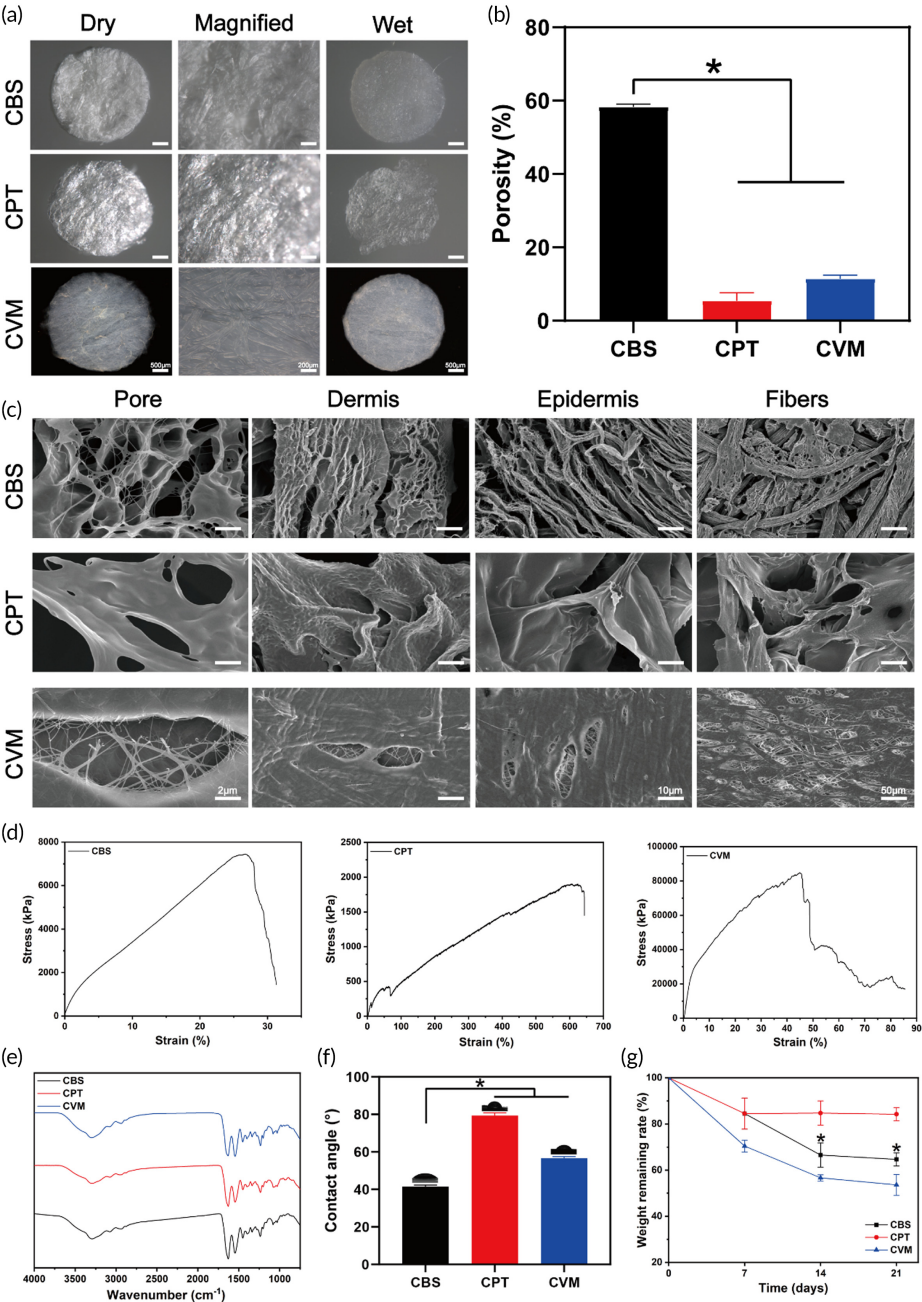
Biological and physicochemical characterization of the CDRS. (a) The stereomicroscope pictures. Scale bar = 500 and 200 μm. (b) The porosity analysis of the CDRS. (c) The morphology of collagen fibers was described by SEM on a 2, 10, and 50 μm, respectively. (d) Representative plots of the mechanical properties for CBS, CPT, and CVM (*n* = 3). Representative images of (e) FT‐IR spectra (n = 3), (f) contact angle, and (g) collagen degradation rate of CBS, CPT, and CVM (*n* = 3). CBS, collagen derived from bovine skin; CDRS, collagen dermal‐replacement scaffolds; CPT, collagen extracted from porcine tendons; CVM, collagen from porcine visceral membrane.

A total of three types of collagen scaffolds are used in biomedicine, which can be involved in the regulation of cell biological activity. To determine the porous and three‐dimensional structure of a scaffold, various analyses are performed. In this study, stereomicroscopy revealed that CBS was more easily infiltrated by PBS or DMEM (Figure 1a). The porosity of CBS was significantly higher (58.28 ± 0.60%, *p* < 0.05) than that of collagen extracted from porcine tendons (CPT) and collagen from the porcine visceral membrane (CVM) (Figure 1b). Correspondingly, SEM also showed that the CBS pores were uniformly distributed. Meanwhile, the CBS collagen fiber arrangement was more directional and its thickness was uniform (Figure 1c). Prior studies have reported that the bovine groups had a markedly higher pore diameter than the porcine groups of the same gender and tissue.^25^ In support, our results showed that more pores were spread over the dermal than the epidermal area in CBS, which can be beneficial for cell adhesion, proliferation, migration, and differentiation.^26^

The dermal substitute for skin wounds should have a certain resistance to compression and hydrophilicity. Therefore, the mechanical properties were also investigated (Figure 1d). We found that the elongation at the break of CBS was the minimum of 31.90 ± 1.45% and the tensile modulus was 285.05 ± 4.61 kPa, which reflected the high parallelism and the strength of collagen fibers in CBS. Previous studies on mechanical properties, the compression stress, and the modulus of porcine collagen sponges were significantly lower relative to those of bovine collagen sponges, which is consistent with our results.^27^ Regarding the effect of tensile modulus on cell infiltration, Gao et al.'s study showed that the tensile modulus of the cross‐linked acellular meniscus scaffold was significantly increased.^28^ The implantation in vivo could induce the growth of autologous cells and tissues to reshape the mechanical structure of the substituted scaffold. The CBS had a more directional arrangement structure of collagen, smaller elongation at the break, and moderately higher tensile modulus, which may play an important role in promoting cell infiltration. With respect to the implications of tensile modulus on scarring, Mikolaszek discussed that DLASil had satisfactory effects on the treatment of scar and keloids in terms of elasticity, higher tensile modulus, softness, and other mechanical adjustable properties.^29^ CBS had a moderately higher tensile modulus that may have contributed to their ability of resistance to stretching and therapeutic effects on wound scar tissues in terms of physical characteristics.

Moreover, Fourier‐transformed infrared spectroscopy (FT‐IR) results showed the main absorption bands of peptide bonds in collagen (Figure 1e), which included N‐H stretching vibration of amide A at 3287 cm^−1^, C=O stretched of amide I at 1630 cm^−1^, N‐H bending of amide II at 1540 cm^−1^, and C‐N stretching of amide III at 1240 cm^−1^. The intensity of this amide band indicated the prevalence of collagen structure, suggesting the interaction of components within the scaffold formation.^30^ The hydrophilicity was reflected by the water contact angle. The contact angle test showed that CBS had a lower angle (41.47 ± 0.70°, *p* < 0.05) than that of CPT (79.33 ± 1.19°) and CVM (56.60 ± 0.67°) (Figure 1f), indicating that CBS had the best hydrophilicity among them.

Finally, we used the lysozyme solution to explore the degradation characteristics of collagen scaffolds. The degradation performance indicated that CVM collagen was the fastest and CPT was the slowest, whereas CBS was in an intermediate state. In fact, the bovine groups indeed exhibited a lower resistance to enzymatic degradation than their porcine counterparts.^31^ The ratio of residual collagen dry weight on Day 21 of CVM was 53.59 ± 5.57% and that of CBS was 64.65 ± 10.35%, both of which were significantly lower than that of CPT (84.24 ± 3.48%, *p* < 0.05) (Figure 1g). This difference may be in view of the freeze‐drying‐generated intermolecular cross‐linking that provides superior organization and stabilization of the helices.^32^ Taken together, these results suggested that CBS had the potential to coordinate the cell biological activity in vitro and the tissue ingrowths for implanting into the full‐thickness skin‐defect wounds.

### 3.2 Morphological characteristics and the biological activity of BADS

The constructed BADS can be considered a complex niche containing MSCs and the surrounding ECM. The ECM biophysically guides cell performance through its three‐dimensional structure and the geometrical placement of the adhesive sites.^33^ The extent to which MSCs are restrictively regulated by ECM may be directly related to the biological processes, including proliferation and differentiation.^34^ More importantly, the cytoskeleton may be the core component of the biological effects of cells under the guidance of mechanical signals.^35^

**FIGURE 2:**
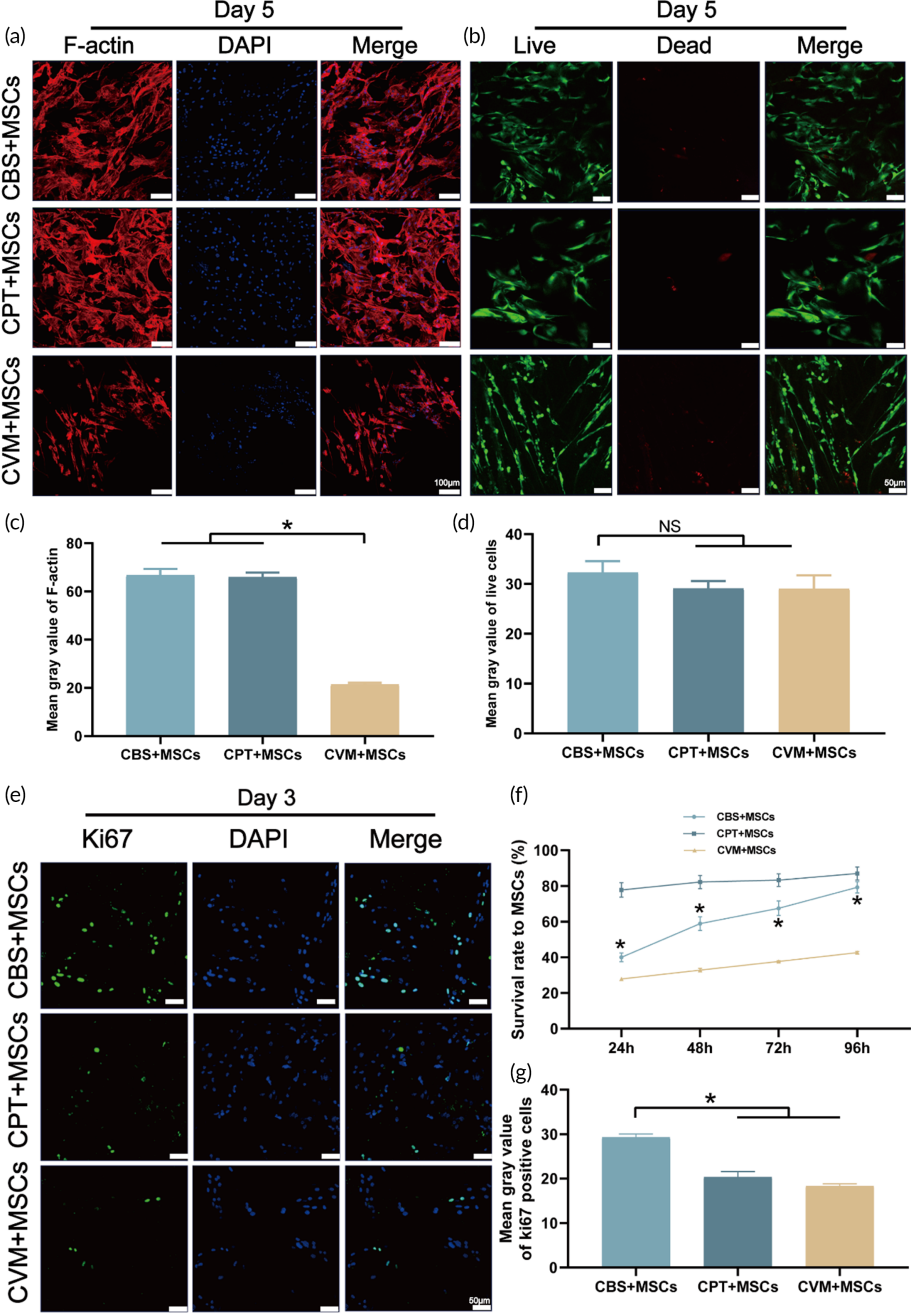
Effects of the CDRS on morphology, viability, and proliferation of MSCs. (a) Effect of CRDS on the cell morphological changes at 5 days. (b) Live/dead staining of MSCs cultured on different collagen scaffolds at 5 days. The quantification analysis of cytoskeleton (c) and live cells (d). (e) Representative Ki67 images of MSCs cultured on CBS, CPT, and CVM at 3 days. (f) CCK8 assay of MSCs viability. (g) Quantitative analyses of ki67‐positive cells. *CBS compared to CPT in panel (f). CBS, collagen derived from bovine skin; CDRS, collagen dermal‐replacement scaffolds; CPT, collagen extracted from porcine tendons; CVM, collagen from porcine visceral membrane; MSC, mesenchymal stem cell.

To determine the cytoskeletal change, BMSCs were stained with phalloidin and DAPI on Day 5 (Figure 2a) and Day 14 (Figure S1) after transplantation to visualize the cell morphology and adhesion. A distinct trend for the increase in F‐actin positive signal was recorded in CBS (66.80 ± 4.48) and CPT (66.00 ± 3.16) than in CVM (21.34 ± 1.26) (*p* < 0.05) (Figure 2c). The cell morphology for CBS and CPT was characterized by the presence of long fusiform, sheet, and filamentous structures. These results suggested that CBS and CPT exhibited significant potential for excellent adhesion. On Day 14, the performance of BMSCs was observed in three‐dimensional space between the different groups (Figure S1). This finding suggested that BMSCs did not grow on the surface of the material, rather they fully extended into the network structure.

To explore the biocompatibility aspect, the live/dead staining assay was performed. The results showed the complete absence of dead cells (red) for all three groups. The mean gray value for the live cells (green) showed no significant difference among the three groups (*p* > 0.05) (Figure 2b,d). Thus, all tested dermal substitutes were excellent biocompatible. To determine the effect of scaffolds on the cell survival ability, the survival rate of BMSCs in different groups was tested using the CCK8 cell count and ki67 staining. As shown in Figure 2f, the survival rate of the CBS and CPT groups was found to be distinctly higher than that of the CVM group. The mean gray value of Ki67 positive cells on the third day (Figure 2e) was recorded to be 29.29 ± 0.74 in CBS, which was significantly higher than that of CPT (20.37 ± 1.21) and CVM (18.33 ± 0.51) (*p* < 0.05) **(**Figure 2g **)**. Therefore, CBS demonstrated a significant potential to promote cell survival and proliferation. This trend was consistent with the growth status of BMSCs scanned in three‐dimensional space on Day 14.

### 3.3 Anti‐inflammatory effect in macrophages by BADS in vitro

**FIGURE 3:**
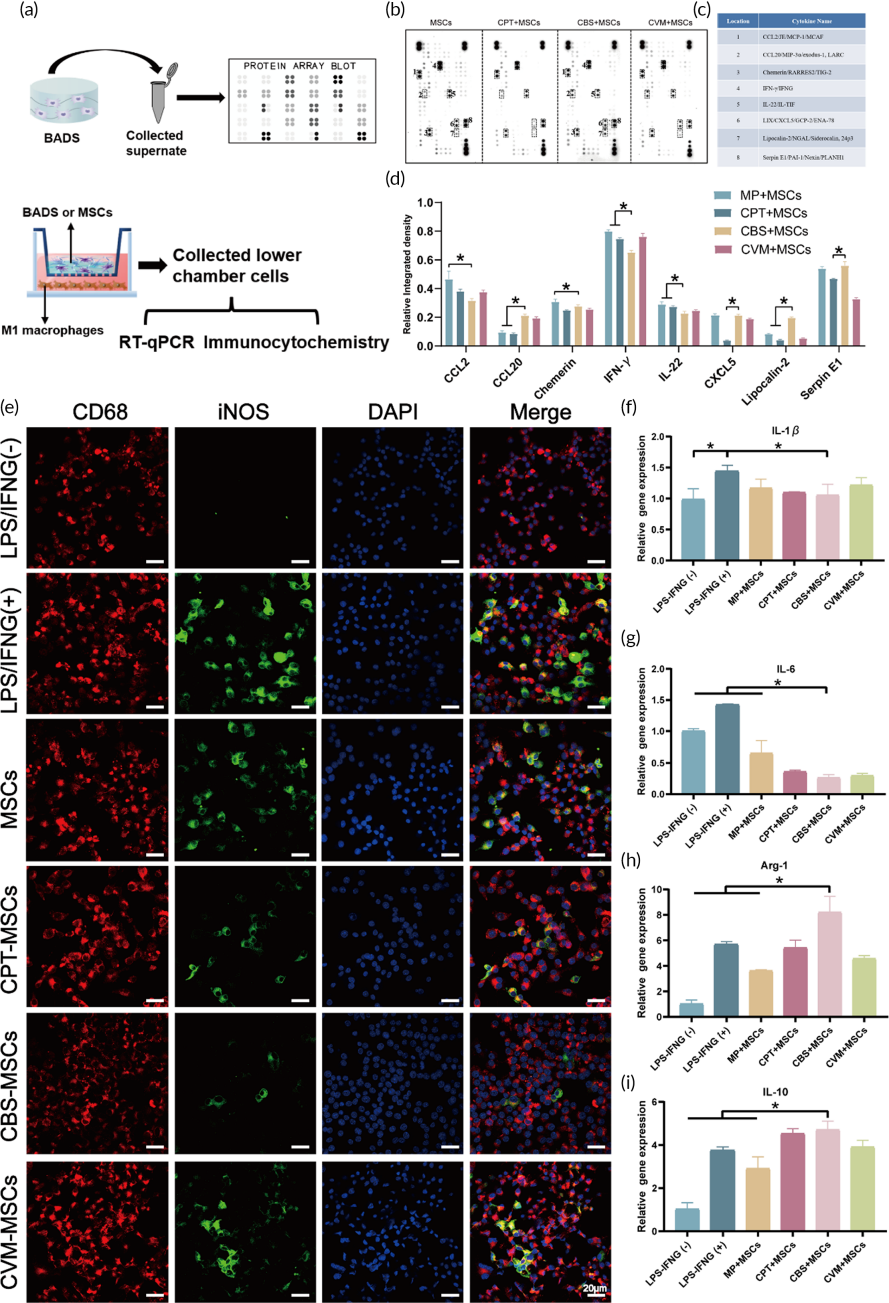
Regulation for paracrine of MSCs and polarization of M1 macrophages under different BADS. (a) A schematic diagram of regulatory experiment. (b) Representative images of protein array blot and (c) the corresponding detected indicator. (d) Quantitative analysis for the related indicator. (e) Representative cell immunofluorescence images of RAW264.7 cells under different treatment conditions. RT‐qPCR analysis of *IL‐1β* (f), *IL‐6* (g), *Arg‐1* (h), and *IL‐10* (i) mRNA expression in RAW 264.7 cells with or without LPS/IFNG, under the intervention of MP‐MSCs, CPT‐MSCs, CBS‐MSCs, and CVM‐MSCs. BADS, biologically active dermal substitutes; CBS, collagen derived from bovine skin; CPT, collagen extracted from porcine tendons; CVM, collagen from porcine visceral membrane; MSC, mesenchymal stem cell.

Recent evidence suggests that collagen scaffolds, a major component of ECM, can induce immune cell activation that triggers specific cytokine responses.^36^ However, the ideal dermal substitute should target the pro‐inflammatory functions of monocytes and macrophages.^37^ To understand whether the inflammatory regulation of collagen scaffold changes after BMSCs implantation, a fundamental analysis of the conditioned media (CM) secretome was performed using a high‐density protein array (Figure 3a, upper panel). Antibody arrays were used for four distinct CM samples obtained from MSCs‐microplate (MP), ‐CPT, ‐CBS, and ‐CVM (Figure 3b). As shown in Figure 3c,d, the relative integrated densities of eight proteins that were differentially produced in four groups on Day 5 post‐transplantation were identified. For lipocalin‐2 (bacteriostatic factor), the CBS‐MSCs group demonstrated an increasing trend relative to those of MP‐MSCs and CPT‐MSCs (*p* < 0.05). The secretome of CXCL‐5 (stimulator of keratinocytes) was also found to be more highly expressed in CBS‐MSCs than in CPT‐MSCs (*p* < 0.05). More importantly, the inflammatory factors, including CCL‐2, chemerin, IFN‐γ, and IL‐22, were decreased by varying degrees in CBS‐MSCs when compared to that in MP‐MSCs (*p* < 0.05).

To further probe the function of targeting inflammation of the products secreted by CM, we co‐cultured MSCs or BADS with M1 macrophages (Figure 3a, lower panel). The mean fluorescence intensity of iNOS (a biomarker of classic‐activated M1 macrophages) was significant in CD68 (a biomarker of unpolarized macrophages) macrophages after stimulation with LPS/IFNG (Figure 3e). In addition, the mean gray value for iNOS was notably lower in CBS‐MSCs than in MP‐MSCs, CPT‐MSCs, and CVM‐MSCs (*p* < 0.05) (Figure S2). Next, we conducted an RT‐qPCR analysis for the induction of inflammatory response in macrophages that manifested as a significant increase in the *IL‐1β* expression (Figure 3f). The increase in the levels of certain inflammatory factors was switched by the CM of the upper chamber. Interestingly, this effect was most significant only in the case of CM from CBS‐MSCs. The *IL‐1β* expression decreased significantly in CBS‐MSCs (1.06 ± 0.18, *p* < 0.05) than in the LPS/IFNG (+) group (1.44 ± 0.10) (Figure 3f). A decrease in the levels of *IL‐6*, a pro‐inflammatory factor, was observed in CBS‐MSCs than in the LPS/IFNG (+) group (*p* < 0.05) (Figure 3g). Anti‐inflammatory factors produced by macrophages, *IL‐10*, and *Arg‐1* revealed an increased level only when treated with CM derived from CBS‐MSCs (*IL‐10*: 4.71 ± 0.40; *Arg‐1*: 8.20 ± 1.24, *p* < 0.05) than from LPS/IFNG (+) and LPS/IFNG (−) (Figure 3h,i). Thus, these results suggested that CBS‐MSCs have a substantial anti‐inflammatory effect and a potential reparatory property.

### 3.4 RNA‐seq and gene set enrichment analysis

**FIGURE 4:**
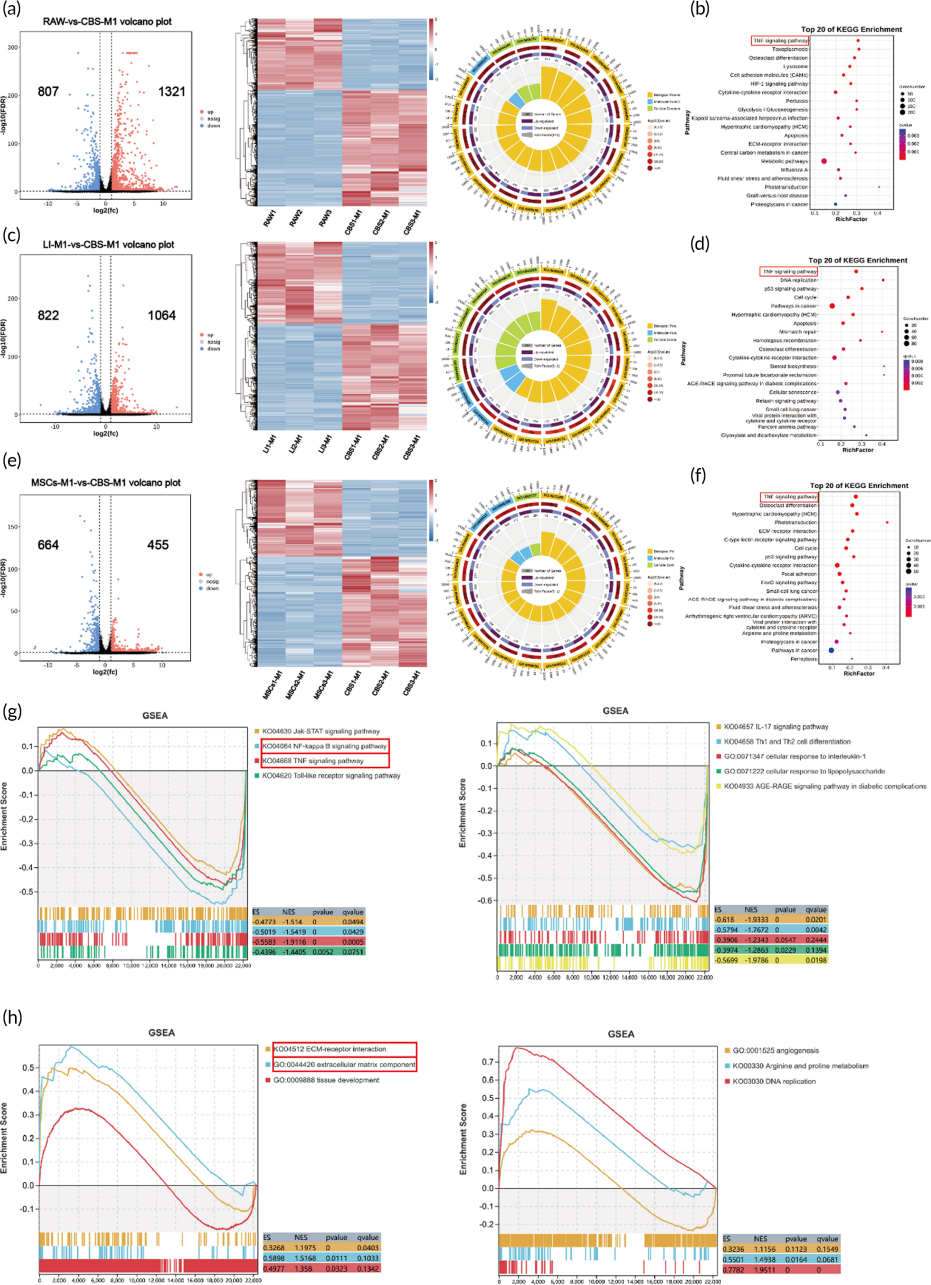
Transcriptomic analysis between CBS‐M1 and LI‐M1. RNA‐seq differential gene analysis is represented by a volcano plot (left sides of a, c, and e), heat map clustering (middle of a, c, and e), and GO (right sides of a, c, and e). KEGG analysis from macrophages treated under different conditions (b, d, and f). GSEA of downregulated gene set (g) and upregulated gene set (h). The red boxes are particularly interesting gene sets. RAW: RAW264.7 cells; Control groups: RAW, LI‐M1, and MSCs‐M1. CBS, collagen derived from bovine skin; CBS‐M1, CBS‐MSCs‐treated M1 macrophages; GSEA, gene set enrichment analysis; IGNG, interferon gamma; KEGG, Kyoto Encyclopedia of Genes and Genomes; LI‐M1, LPS/IFNG‐activated M1 macrophages; LPS, lipopolysaccharide; MSCs‐M1, MSCs‐treated M1 macrophages; MSC, mesenchymal stem cell.

Based on the abovementioned experimental results, we believe that CBS‐MSCs played a critical role in regulating M1 macrophage polarization and inhibiting inflammation. To date, only a few studies have reported the gene expression profile of biologically modified collagen scaffolds in remodeling macrophages.^38^ To further clarify the underlying molecular mechanism, RNA‐seq and gene set enrichment analysis (GSEA) were performed to comprehensively and systematically reveal how CBS‐MSCs regulate M1 macrophages. We first selected a gene with adj. *p*‐value <0.05 and |log2FC| > 1 as significantly different. The results indicated that a total of 1321 genes were upregulated and 807 genes were downregulated in the CBS‐MSCs‐treated M1 macrophages (CBS‐M1) when compared with RAW264.7 (RAW). Enrichment of the top 20 Gene Ontology (GO) terms mainly revealed a biological process (e.g., GO: 0031347, 0071345, and 0034097) as well as cell components (GO: 00005737 and 0044444) (Figure 4a). Kyoto Encyclopedia of Genes and Genomes (KEGG) (Figure 4b) analysis showed the TNF signaling pathway as the main enrichment pathway. A total of 1064 upregulated genes and 822 downregulated genes were profiled in CBS‐M1 when compared with that in LPS/IFNG‐activated M1 macrophages (LI‐M1). GO enrichment was dominated by the biological processes (e.g., GO: 0009628, 0033554, and 0071310), whereas others included molecular functions (GO: 0005515 and 0005488) and cellular components (GO: 0005737, 0005622, and 0044424) (Figure 4c). The TNF signaling pathway was also the main signaling pathway enriched by KEGG (Figure 4d). When compared with the MSCs‐treated M1 macrophages (MSCs‐M1), there were 455 upregulated genes and 664 downregulated genes in CBS‐M1. The GO term such as GO: 0072359, 0042127, and 0008283, and other biological processes occupied the main part (Figure 4e). KEGG enrichment showed that, except for the TNF signaling pathway, focal adhesion was also important in the interaction between ECM and cellular (Figure 4f).

As LI‐M1 could simulate inflammation in vitro, CBS‐M1 and LI‐M1 were selected for GSEA. When compared with the LI‐M1, the related inflammatory gene sets including genes of the NF‐κB signaling pathway (NES = −1.54, *p* < 0.05), TNF signaling pathway (NES = −1.91, *p* < 0.05), JAK–STAT signaling pathway (NES = −1.51, *p* < 0.05), and toll‐like receptor signaling pathway (NES = −1.44, *p* < 0.05) were significantly downregulated in CBS‐M1 (Figure 4g). When combined with the GSEA results and the existing research, it was noted that the intracellular conduction pathway of the TNF‐α signal can be divided into NF‐κB, JNK, and apoptosis. Therefore, we concluded that CBS‐MSCs may play an anti‐inflammatory role via the TNF‐α/NF‐κB signaling axis.^39^

CBS‐MSCs may also regulate immunoreaction, such as the IL‐17 signaling pathway (NES = −1.93, *p* < 0.05), Th1 and Th2 differentiation (NES = −1.77, *p* < 0.05), and cellular responses to IL‐1 (NES = −1.23, *p* < 0.05), which were significantly reduced in CBS‐M1 enrichment (Figure 4g). In contrast, some signaling pathways related to cell proliferation and tissue growth were significantly increased in CBS‐M1, such as ECM‐receptor interaction (NES = 1.20, *p* < 0.05), ECM components (NES = 1.52, *p* < 0.05), tissue development (NES = 1.36, *p* < 0.05), and angiogenesis (NES = 1.12, *p* = 0.11) (Figure 4h). Therefore, in terms of the gene expression profile, CBS‐MSCs played an important role in anti‐inflammation as well as promoted cell proliferation and ECM remodeling.

### 3.5 Inflammatory regulation affected by the TNF‐α/NF‐κB signaling pathway in CBS‐M1

**FIGURE 5:**
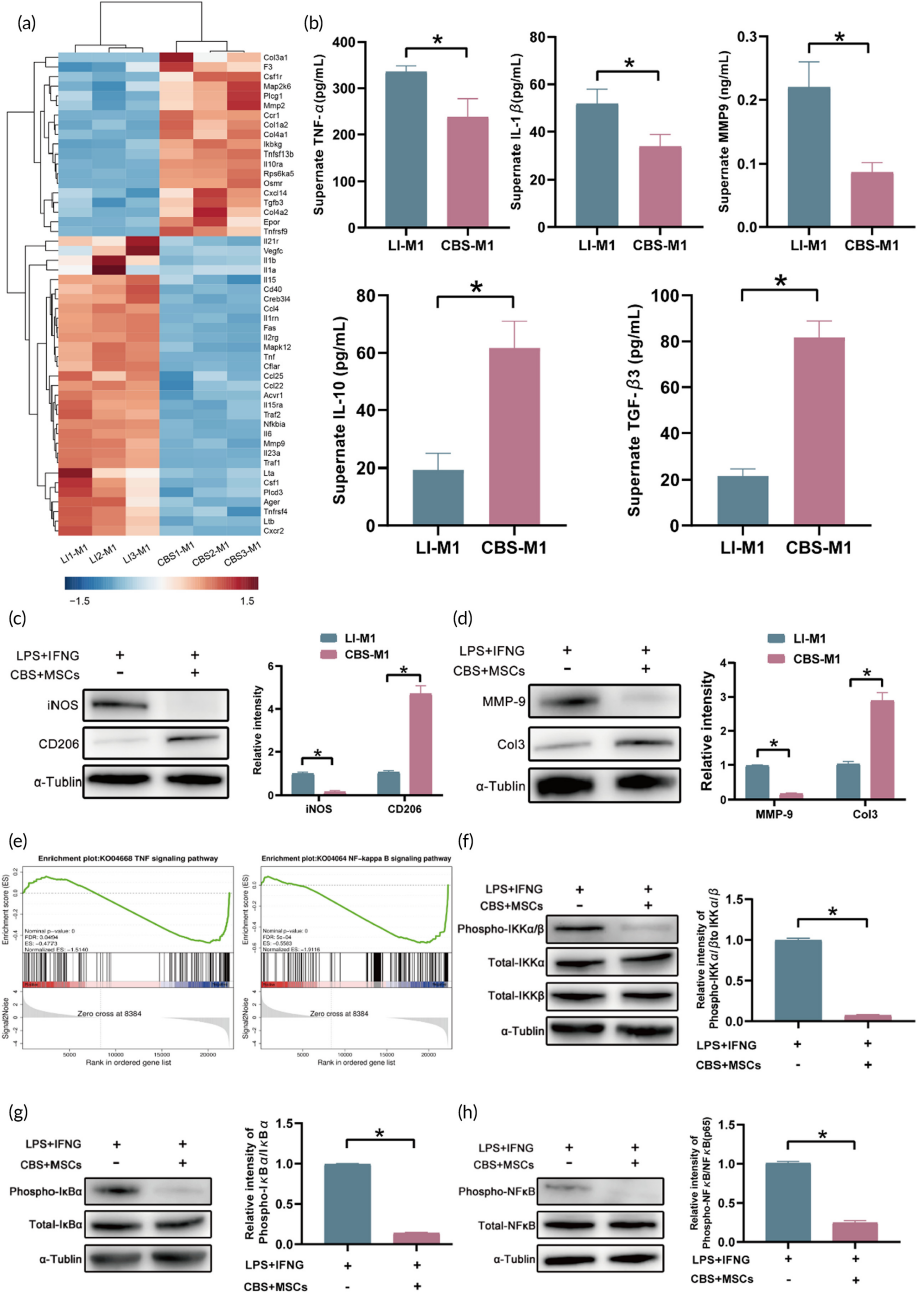
The potential mechanism for the modulation of inflammatory response in M1 macrophages by CBS‐MSCs. (a) Heatmap depicting the expression of inflammatory‐related genes. (b) ELISA assay for the detection of TNF‐α, IL‐1β, MMP‐9, IL‐10, and TGF‐β3. The expression and quantification of (c) iNOS and CD206 and (d) MMP‐9 and Col3 at the protein levels. (e) GSEA analysis of the TNF‐α and NFκB signaling pathways. Expression change of (f) phospho‐IKKα/β and total IKKα/β, (g) phospho‐IκB and total IκB, and (h) phospho‐NFκB and total NFκB at the protein levels. CBS, collagen derived from bovine skin; GSEA, gene set enrichment analysis; MSC, mesenchymal stem cell.

To reveal the molecular mechanism involved in regulating inflammation in M1 macrophages treated with CBS‐MSCs, we performed a distinct cluster analysis of the Top50 genes associated with CBS‐M1 and those with LI‐M1 (Figure 5a). As shown in the figure, the pro‐inflammatory mediators of CBS‐M1, such as *Il1b*, *Il15*, *Ccl4*, *Tnf*, *Il6*, *Mmp9*, and *Il23a*, were significantly downregulated when compared with those of LI‐M1, whereas the anti‐inflammatory factors, such as *Il10ra*, *Cxcl14*, and *Tgfb3*, were significantly upregulated. Genes associated with ECM production, such as *Col3a1* (Top1), *Col1a2*, *Col4a1*, and *Col4a2*, were also significantly upregulated in CBS‐M1. The components of the TNF‐α/NF‐κB signaling pathway, such as *Tnf*, *Traf2*, *Nfkbia*, *Traf1*, and *Tnfrsf4*, were significantly downregulated in CBS‐M1 than in LI‐M1. This observation supported our hypothesis that CBS‐MSCs play an anti‐inflammatory role via the TNF‐α/NF‐κB signaling pathway.

We further investigated the expression of the corresponding differential genes. ELISA results showed that (Figure 5b) pro‐inflammatory mediators including TNF‐α (237.60 ± 39.89 pg/ml), IL‐1β (33.75 ± 5.09 pg/ml), and MMP‐9 (0.09 ± 0.02 ng/ml) levels were significantly lower in CBS‐M1 than those of LI‐M1 (*p* < 0.05). On the contrary, the levels of anti‐inflammatory factors IL‐10 (61.72 ± 9.33 pg/ml) and TGF‐β3 (81.71 ± 7.18 pg/ml) were much higher in CBS‐M1 than in LI‐M1 (*p* < 0.05). Western blotting results revealed (Figure 5c) that the relative iNOS level in CBS‐M1 was significantly decreased (0.15 ± 0.01, *p* < 0.05) when compared with that in the control group. The relative CD206 (a biomarker for M2 macrophages in alternative pathways) level was significantly increased (4.70 ± 0.38, *p* < 0.05) in CBS‐M1. Interestingly (Figure 5d), the MMP‐9 level, a matrix metalloproteinase that degrades ECM, in CBS‐M1 was also decreased (0.17 ± 0.01, *p* < 0.05). The Col3, which can promote the formation of granulation tissues and connective tissue matrix, showed an opposite trend of MMP‐9 in CBS‐M1 (2.88 ± 0.24), which was significantly higher than that in LI‐M1 (1.03 ± 0.07, *p* < 0.05). However, when compared with a hypertrophic scar on a normal wound, we paid more attention to whether sufficient collagen was present to fill the tissue defects in chronic diabetic wounds.^40^, ^41^ Based on these results, we speculated that M1 macrophages in vivo after CBS‐MSCs treatment may reverse scar healing to varying degrees through a paracrine action (significantly reduced MMP‐9) or direct participation in wound remodeling (significantly increased type‐III collagen) in the early stage of chronic diabetic wounds.

Combined with the abovementioned phenotypic changes, we performed a GSEA to investigate whether the TNF‐α/NF‐κB pathway was affected by CBS‐MSCs **(**Figure 5e **)**. As expected, the phospho‐IKK α/β (Ser176/180) levels in CBS‐M1 were significantly reduced when compared with those in the control group. The ratio of phospho‐IKK α/β and total IKKα/β was also significantly reduced in the treatment group (0.07 ± 0.01, *p* < 0.05) (Figure 5f). Similarly, the ratios of phospho‐IκB/total IκB and phospho‐NFκB/total NFκB were significantly reduced in CBS‐M1 (0.14 ± 0.01 and 0.25 ± 0.02, respectively, *p* < 0.05) (Figure 5g,h). Generally, the IκB component of the NFκB and IκB complex was degraded by phosphorylated IKKβ, resulting in NFκB translocation into the nucleus and the induction of pro‐inflammatory gene transcription.^42^ Nonetheless, CBS‐MSCs may inhibit this process by reducing the secretion of cytokines such as TNF‐α or IL‐1β.^43^ Similar to our results, Ding et al. showed that propofol inhibited TNF‐α‐induced MMP‐9 expression and the degradation of type IV collagen in hCMEC/D3 cells by suppressing the Ca^2+^/CAMKII/ERK/NF‐κB signaling pathway.^44^ Therefore, we concluded that CBS‐MSCs could negatively regulate inflammation by affecting the TNF‐α/NF‐κB signaling pathway.

### 3.6 Analysis of wound regeneration induced by BADS

**FIGURE 6:**
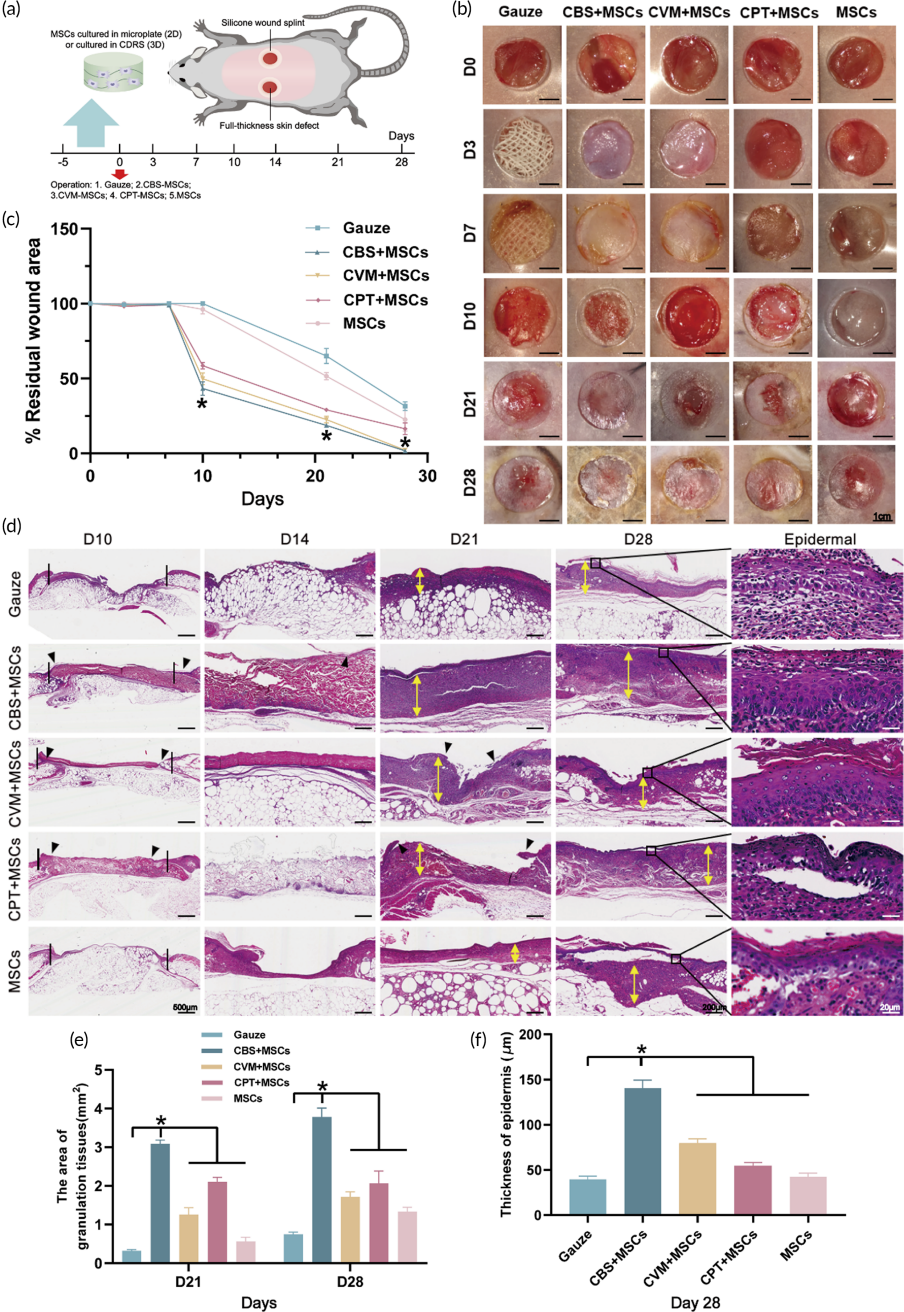
Investigation of back wound healing in db/db mice. (a) A schematic representation of the preparation and operation for implantation. (b) Representative images of the wound areas. (c) The quantification of the residual wound area. (d) H&E staining of the implanted sections collected at indicated time points, illustrating wound gap at Day 10 (black lines), host tissue migration (black triangles), the thickness of the granulation tissues (double‐headed yellow arrows), and epidermal tissues at Day 28 (black squares). Quantitative analysis of the area of the granulation tissues (e) and the thickness of the epidermis (f).

The polarization disorder of macrophages is an important cause of continued inflammation in chronic diabetic wounds, which mainly manifests as a significantly increased proportion of M1 macrophages.^45^ The abovementioned experiments proved that CBS‐MSCs may remodel the function of M1 macrophages by influencing the TNF‐α/NF‐κB signaling pathway. To understand the therapeutic efficacy of BADS, the back wound of db/db mice was assessed. The surgical process of full‐thickness skin defect is shown in Figure 6a. BADS were cultured in vitro for 5 days in advance and then transplanted into the back wound of the db/db mice. Over time, BADS displayed different pro‐healing effects (Figure 6b). From Days 0 to 7, no difference was recorded in the residual wounds among the groups; however, the exudate in the control (gauze) group had increased significantly. Previous studies also showed that the time of chronic wound healing in db/db mice was significantly longer than that of normal wounds.^46^ Until Day 10, new granulation tissues (GTs) and epithelialization degrees were significantly increased in CBS‐MSCs when compared with those in the other groups. The residual wound area (43.26 ± 4.60%) on Day 10 in CBS‐MSCs was significantly lower than that in the control (100.10 ± 0.05%, *p* < 0.05) (Figure 6c). On Day 21, the wound in the CBS‐MSCs group was epithelialized. The wound closure rate (88.54 ± 2.70%) was significantly higher than that of the control group (49.90 ± 1.10%, *p* < 0.05) (Figure S3). By Day 28, the CBS‐MSCs group was fully epithelialized. The wound closure rate (97.99 ± 2.14%) was significantly higher and the residual wound area (1.66 ± 0.30%) was lower in CBS‐MSCs than those in the control and MSCs groups (*p* < 0.05) (Figures 6c and S3).

To further understand the structural and functional changes of BADS in promoting wound healing, H&E staining was performed (Figure 6d). Morphological properties, such as thickness, texture, and porosity, of the dermal substitutes covered on the wounds, were observed on Days 10 and 14. Reparative cells also migrated into the collagen scaffolds between the normal skin and wound edge (indicated by black triangles). Thus, we demonstrated that the three types of biological materials had excellent biocompatibility. Regarding the degradation time of BADS, although we found degradation of the central base part of the collagen scaffolds where epithelialization and gradually thickening of the wound could be seen (starting from the 18th day), the upper part was still not degraded. This phenomenon continued until the wound was completely healed. Restricted by the size of the wounds and the limited time to observe, we could not know the precise time of complete degradation for collagen. We will definitely consider this issue in the future.

In chronic diabetic wounds, the lack of GTs may be the direct cause of weak epithelial structure and dermal tissue ulceration.^47^ On Day 21, the GT area in the CBS‐MSCs group was 3.09 ± 0.09 mm^2^, which was significantly higher than that in the control group (0.38 ± 0.07 mm^2^, *p* < 0.05) and in the other 3 groups (CVM‐MSCs: 1.26 ± 0.17 mm^2^; CPT‐MSCs: 2.11 ± 0.11 mm^2^; MSCs: 0.73 ± 0.24 mm^2^, *p* < 0.05) (Figure 6e). On Day 28, the area of regenerated GTs in CBS‐MSCs continued to increase (4.18 ± 0.18 mm^2^), which was significantly higher than that of the control group (0.75 ± 0.06 mm^2^, *p* < 0.05). Then, we analyzed the area of scar tissues with reference to the research of Zhou and Li^48^, ^49^; the scar area of CBS‐MSCs on Day 28 was 7.78 ± 0.66 mm^2^, which was significantly lower than that of CPT‐MSCs (21.57 ± 1.28 mm^2^) and MSCs groups (10.50 ± 0.26 mm^2^) (*p* < 0.05) and marginally higher than that of gauze (4.54 ± 0.41 mm^2^) and CVM‐MSCs (5.72 ± 0.39 mm^2^) (*p* < 0.05) (Figure S4). Next, we compared the skin thickness in the control group (39.60 ± 3.44 μm) and found that the skin thickness in the CBS‐MSCs group was significantly increased (140.5 ± 8.91 μm, *p* < 0.05) (Figure 6f). This result was more favorable for the physiologic repair of chronic diabetic wounds and might reduce the probability of ulcer recurrence after healing.

### 3.7 Investigation of macrophage polarization in vivo

**FIGURE 7:**
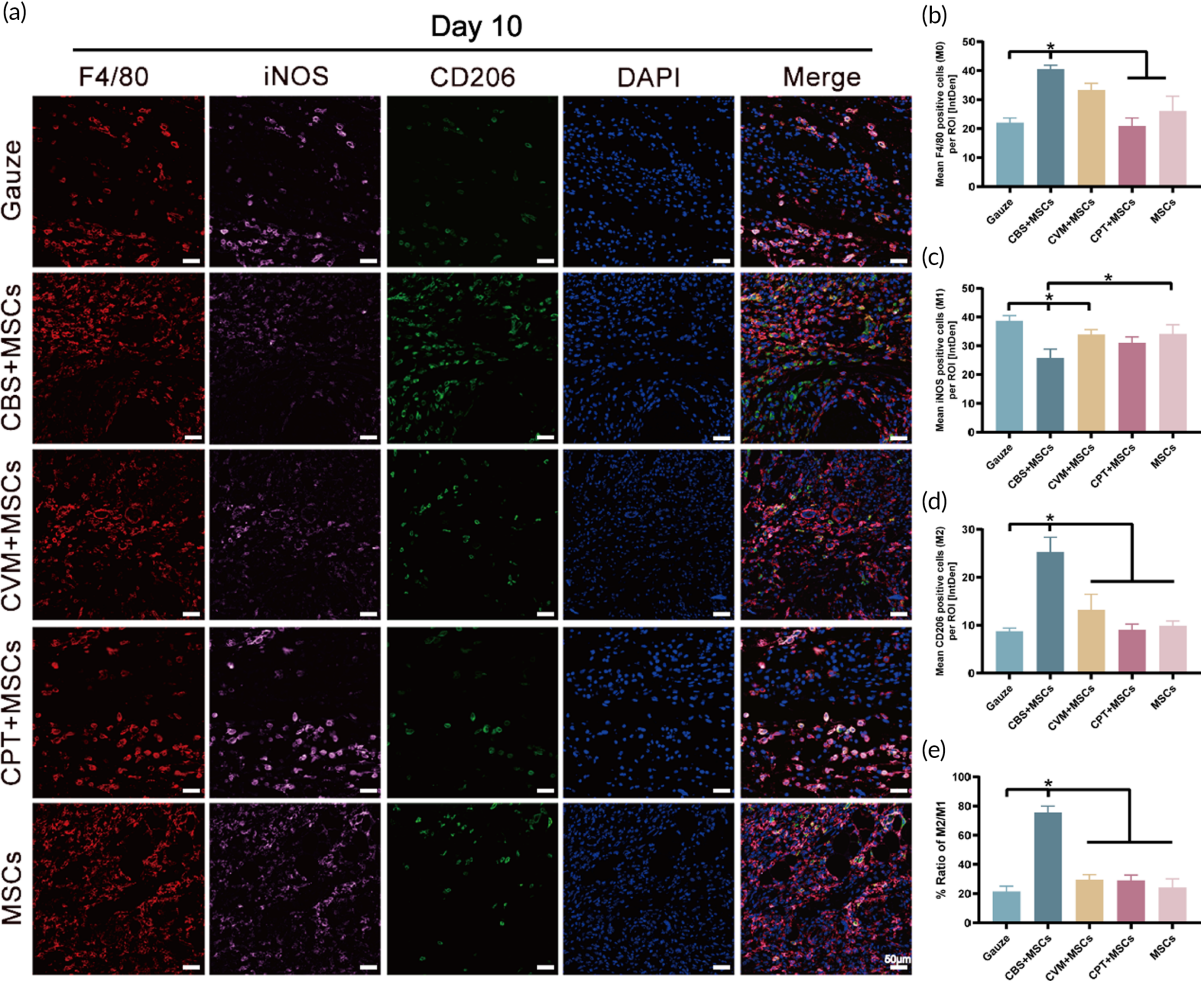
Regulation of polarization of wound macrophages by BADS in db/db mice. (a) Representative immunofluorescence images of M0 macrophages, M1 macrophages, and M2 macrophages in the wound tissues at Day 10 post‐wounding. Quantification of (b) M0 macrophages, (c) M1 macrophages, and (d) M2 macrophages. (e) The ratio of M2/M1 macrophages. BADS, biologically active dermal substitutes.

At 5–7 days after a skin injury, most M1 macrophages around the wound is be replaced by M2 macrophages, which promote angiogenesis, keratinocytes, and fibroblast proliferation.^50^ Hence, localized and properly controlled inflammation is a potential trigger for proliferation and remodeling.^51^ To evaluate this “trigger factor,” we selected the wound tissues on Day 10 to study the differences in macrophage polarization. Multiplex fluorescence immunohistochemical analysis (Figure 7a–d) showed that the average fluorescence intensity of iNOS‐positive cells in the control group was 38.69 ± 1.78 and that of CD206‐positive cells was 8.74 ± 0.69. The M2/M1 ratio was only 21.43 ± 3.81%, which was consistent with past results that about 80% of the cells at the edge of DFUs were M1 macrophages.^52^ Similar to the non‐activated macrophages (M0) in the tissues (Figure 7a,b), a sufficient number and normal functions of M0 formed the basic parameters to ensure their phagocytosis, chemotaxis, inflammation, and proliferation.^53^ We found that the average fluorescence intensity of F4/80‐positive (a biomarker of unpolarized macrophages) cells in the CBS‐MSCs group was 40.52 ± 1.38, which was significantly higher than that in the control, CPT‐MSCs, and MSCs groups (*p* < 0.05). This finding was beneficial for CBS‐MSCs to play a regulatory role to some extent in macrophage development. Considering the regulation of M1 macrophages, an in vitro experiment was conducted, which showed that CBS‐MSCs could inhibit the secretion of inflammatory factors and the expression of the *iNOS* via the TNF‐α/NF‐κB signaling pathway, thereby inhibiting M1 macrophage polarization. In vivo data also showed (Figure 7a,c) that the average fluorescence intensity of the M1 cells in CBS‐MSCs (25.72 ± 3.13) was significantly lower than that in the control (38.69 ± 1.78, *p* < 0.05) and MSCs groups (34.15 ± 3.17, *p* < 0.05).

RNA‐seq results showed that the CXCL14 expression was significantly increased in CBS‐M1 than in LI‐M1 (Figure 5a). Brown adipose tissues secrete CXCL14 under thermogenic activation, which further promotes the polarization and recruitment of M2 macrophages and induces the transformation of white adipose tissues into brown adipose tissues.^54^ This observation suggested that CXCL14 may also drive M2 macrophage polarization in chronic diabetic wounds under CBS‐MSCs. As expected, CBS‐MSCs could promote M2 macrophage polarization in vivo (Figure 7a,d). The average fluorescence intensity of M2 cells in CBS‐MSCs on Day 10 was 25.30 ± 3.05, which was significantly higher than that of the control and other three groups (*p* < 0.05). M2/M1 proportion in the CBS‐MSCs group was 75.61 ± 4.36%, which was significantly higher than that in the control group (21.43 ± 3.81%, *p* < 0.05) (Figure 7a,e). Hence, CBS‐MSCs could promote polarization of M2 macrophages in chronic diabetic wounds, which might consequently modulate the proliferation and differentiation of reparatory cells for optimal wound healing.

### 3.8 Inhibition of pro‐inflammatory factors

Macrophages activated by the typical pathways (M1) are highly phagocytic and can effectively produce pro‐inflammatory cytokines, namely IL‐1α, IL‐1β, IL‐6, and TNF.^55^ In contrast, a lower absolute number of M2 macrophages and a higher M1:M2 macrophage ratio in the wound decreases anti‐inflammatory cytokines including IL‐10, TGF‐α, and TGF‐β.^56^ The abovementioned cytokines regulated inflammation and induced proliferation, respectively.

**FIGURE 8:**
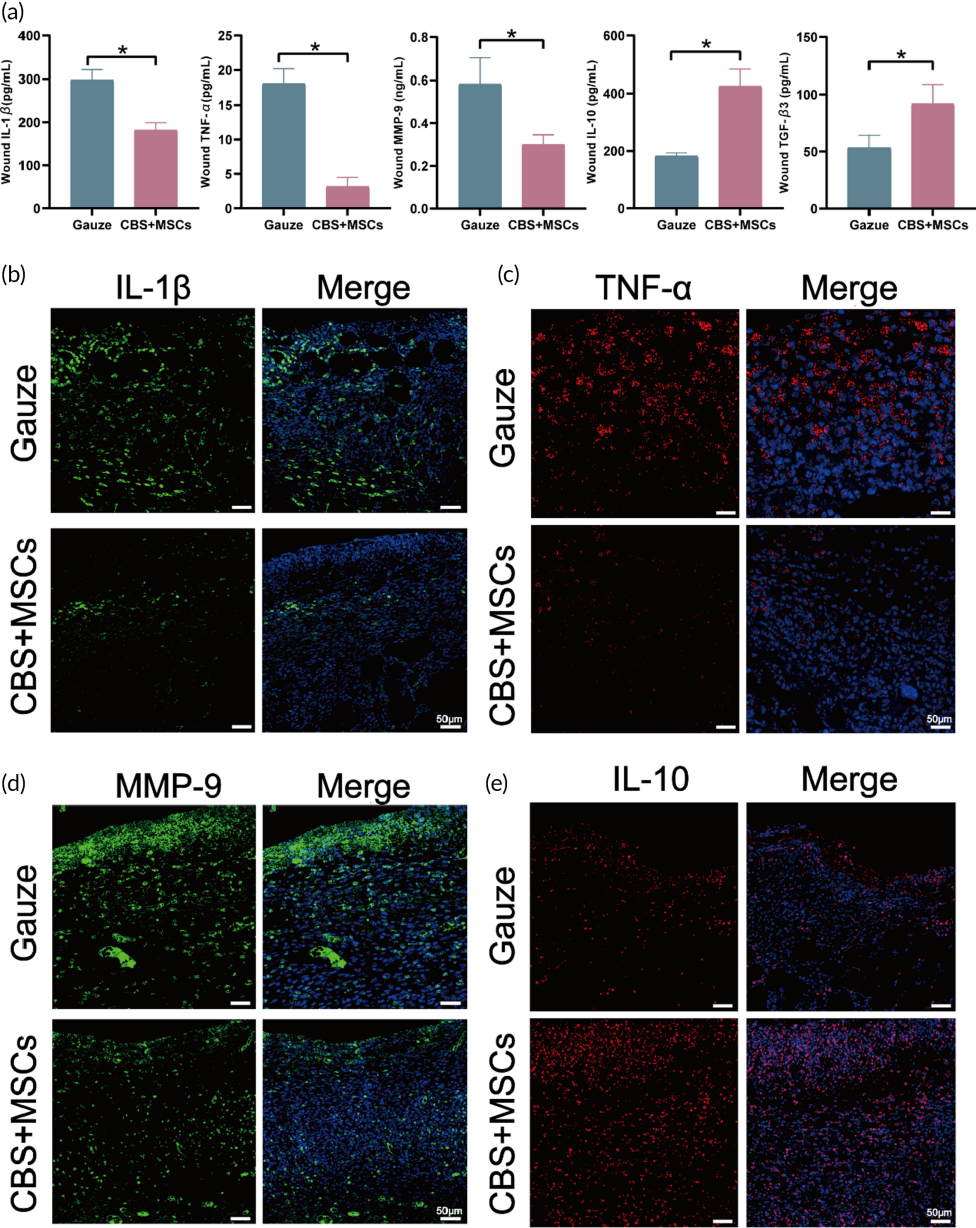
Analysis of the effects of CBS‐MSCs on cytokines in vivo according to the RNA‐seq results. (a) ELISA for the detection of IL‐1β, TNF‐α, MMP‐9, IL‐10, and TGF‐β3 at the wound sites on Day 10. Immunofluorescence staining of (b) IL‐1β, (c) TNF‐α, (d) MMP‐9, and (e) IL‐10. CBS, collagen derived from bovine skin; MSC, mesenchymal stem cell.

Based on this observation, when combined with the transcriptome‐sequencing results, the corresponding in vivo inflammatory mediators and anti‐inflammatory factors were analyzed. IL‐1β secretion in the wound implanted with CBS‐MSCs was 181.70 ± 17.33 pg/ml, which was significantly lower than that in the control group (298.40 ± 22.73 pg/ml, *p* < 0.05) (Figure 8a). The mean fluorescence intensity of IL‐1β around the wound (6.71 ± 1.05) was significantly lower than that of the control group (18.80 ± 0.82, *p* < 0.05) (Figures 8b and S5a). In this study, the two pro‐inflammatory factors (MMP‐9: 0.30 ± 0.05 ng/ml; TNF‐α: 3.17 ± 1.33 pg/ml) were significantly lower than those in the control group (*p* < 0.05) (Figure 8a), and the corresponding average fluorescence intensity showed a consistent difference (Figures 8c,d and S5b,c). The decreased secretion of these pro‐inflammatory factors, especially MMP‐9, may improve the dermal three‐dimensional collagen structure by recovering the function of fibroblasts and myofibroblasts.^57^

Considering the anti‐inflammatory factor IL‐10, its concentration in CBS‐MSCs wound tissues were 425.20 ± 57.92 pg/ml, which was 2.3 times higher than that in the control group (183.40 ± 10.25 pg/mL, *p* < 0.05). The average fluorescence intensity was 4.7 times that of the control group (19.28 ± 0.94 vs. 4.13 ± 0.20, *p* < 0.05) (Figures 8e and S5d). TGF‐β3 is an important marker for scar reduction in wound healing. In this study, the TGF‐β3 concentration in wound tissues containing CBS‐MSCs was 92.06 ± 16.91 pg/ml, which was significantly higher than that in the control group (*p* < 0.05) (Figure 8a). Therefore, we hypothesized that CBS‐MSCs successfully shifted the “trigger factor” to a side favorable for wound healing during proliferation. CBS‐MSCs may play a dual role in inhibiting pro‐inflammatory factors and promoting anti‐inflammatory mediators.

### 3.9 Neovascularization and collagen‐deposition analysis

**FIGURE 9:**
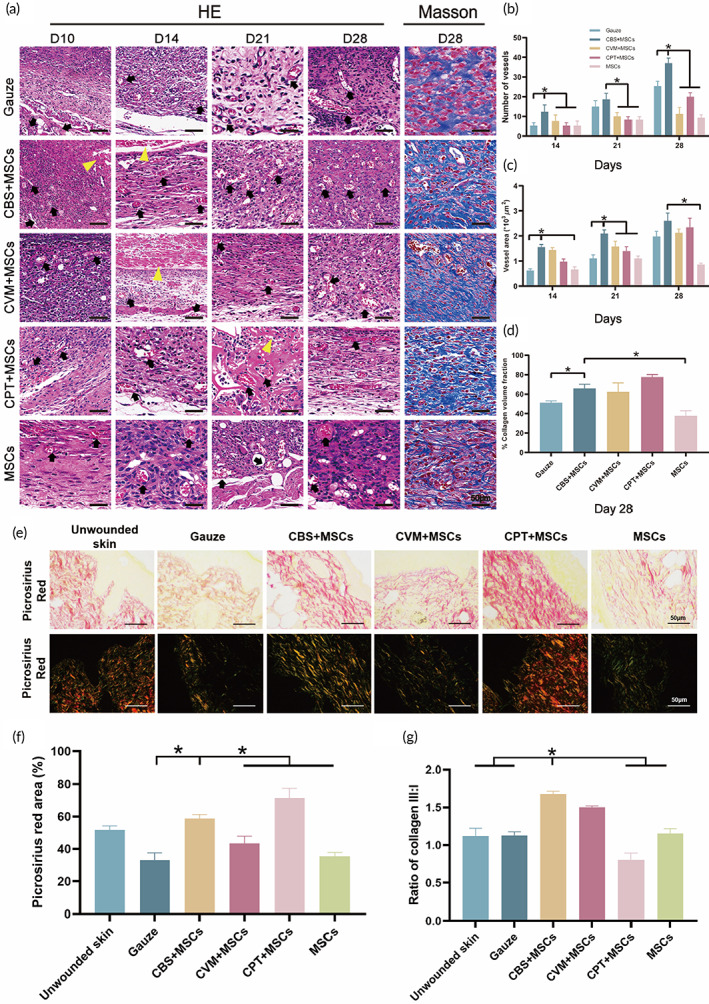
Studies on angiogenesis and collagen deposition. (a) The H&E staining of capillary densities (black arrows) and implanted BADS (yellow triangles) on Days 10, 14, 21, and 28. Masson's trichrome staining of collagen fibers at the wound sites. (b) The quantification of microvessels' calculation, (c) microvessels areas, and (d) collagen volume fraction. (e) Collagen disposition was analyzed using Picrosirius red staining under a light (collagen: red) and polarized light (collagen: yellow‐orange to green birefringence) microscope. (f) Quantitative analysis of picrosirius red staining. (g) Ratio of collagen III:I in each group. BADS, biologically active dermal substitutes.

Neovascularization is an important sign of cells entering the proliferation stage, and the reduced ability of endothelial progenitor cells to form new blood vessels is an important reason for the interruption of re‐epithelialization in chronic diabetic wounds.^58^, ^59^ To understand the effects of BADS on angiogenesis, the number and area of microvessels were analyzed on Day 10 when the changes in wound healing were most evident. No significant differences were detected between the groups (Figure 9a). On Day 14, the number of microvessels in CBS‐MSCs (12.33 ± 3.51) was significantly higher than that in the control and other groups (*p* < 0.05) (Figure 9b). The number of microvessels in CBS‐MSCs (18.67 ± 3.06) on Day 21 did not significantly increase when compared with that in the control group (*p* > 0.05); however, it was higher than that in the other three treatment groups (*p* < 0.05). Consistent with the observations made on Day 14, the number of microvessels in CBS‐MSCs reached the highest value (37.00 ± 2.65) on Day 28, which was 1.5–4.2 times higher than that in the other groups. In addition, we evaluated the microvascular area (Figure 9c) and found that the microvascular area of CBS‐MSCs was significantly larger than that of the control and MSC groups on Days 14 and 21. The maximum value was 2.60 ± 0.31 × 10^3^ μm^2^ on Day 28, which was significantly higher than that of MSCs (0.86 ± 0.06, *p* < 0.05) × 10^3^ μm^2^.

The main reparative cells filled with wounds, fibroblasts, and myofibroblasts, cannot synthesize the ECM under the microenvironment of chronic diabetic wounds, which resulted in an imbalance in the deposition and degradation of collagen.^60^ To compare the effect of BADS on collagen generation, Masson staining was performed on the fully healed wound on Day 28 (blue staining indicated collagen fiber) (Figure 9a). We displayed that a dense arrangement of collagen fiber was present in CBS‐MSCs with a larger diameter than that of the control and MSCs groups. The quantitative analysis of collagen volume fraction in each group **(**Figure 9d) also supported this result, indicating that the collagen volume fraction of CBS‐MSCs on Day 28 (65.97 ± 4.25%) was significantly higher than that of the control and MSCs groups (51.14 ± 1.90% and 37.89 ± 4.99%, respectively, *p* < 0.05). As a result, CBS‐MSCs could greatly promote angiogenesis and improve collagen tissue deposition for the physiological healing of chronic diabetic wounds.

To investigate the alignment of collagen fibers between normal unwounded skin and samples from each group, Picrosirius red was performed on Day 28. Collagen fibers were studied under light and polarized light microscopy according to Xin and Qin et al.^61^, ^62^ First, collagen deposition in unwounded skin was sparse, with a trend toward a basket weave orientation (Figure 9e). This phenomenon was consistent with the finding of a study by Jorgensen et al.^63^ Picrosirius red staining of unwounded skin showed that the ratio of collagen area accounted for 51.67 ± 2.52% under bright‐field (Figure 9f). The collagen was mainly composed of type I collagen (stained orange/red color) under polarized light. Second, the collagen area ratio of CPT‐MSCs (71.33 ± 6.11%) was significantly higher than that of CBS‐MSCs (*p* < 0.05), which showed thick fibers bundle and dense collagen deposition. The collagen III/I ratio was 0.81 ± 0.09 (Figure 9g), indicating that CPT‐MSCs was mainly composed of collagen I. The red‐stained collagen bundles of CBS‐MSCs showed a basket wave orientation (Figure 9e). Compared with unwounded normal skin, CBS‐MSCs had lightly aligned collagen fibers, but the difference was not significant in the area of red‐stained collagen (*p* > 0.05). However, collagen III (stained green color) of CBS‐MSCs was more distinct (III/I collagen ratio: 1.68 ± 0.04) compared to the other groups, which was consistent with our findings in vitro. Finally, similar to what was found using Masson staining, collagen area ratio of CBS‐MSCs (58.57 ± 2.49%) was significantly larger than that of Gauze (33.00 ± 4.58%), CVM‐MSCs (43.33 ± 4.51%), and MSCs (35.33 ± 2.51%) (*p* < 0.05). These results indicated that the collagen deposition of CBS‐MSCs in db/db mice back wounds was analogous to that in normal unwounded skin. The increase of type III collagen and ECM could reduce the ulcer recurrence of chronic diabetic wounds after healing.

## 4 CONCLUSION

In this study, we confirmed that CBS has superior physicochemical properties, mechanical properties, and biocompatibility. The established CBS‐MSCs exhibited certain characteristics of anti‐inflammatory paracrine action. The interaction with M1 macrophages showed that CBS‐MSCs could inhibit the polarization of pro‐inflammatory macrophages. Transcriptome sequencing revealed that CBS‐MSCs played a role in inhibiting pro‐inflammatory factors, matrix metalloproteinases, and promoting anti‐inflammatory mediators and collagen function. This phenomenon may be achieved by affecting the TNF‐α/NF‐κB signaling pathway to remodeling the function of M1 macrophages. CBS‐MSCs could also achieve noncontractile wound healing in db/db mice. CBS‐MSCs‐mediated macrophages could also be polarized in the M2‐type direction in vivo. The related cytokines displayed a consistent trend in vitro. The GT area and epidermal thickness were increased, indicating that CBS‐MSCs possess the clinical transformation potential to improve the recurrence of chronic diabetic wounds from the pathological perspective of wound healing.

## AUTHOR CONTRIBUTIONS

**Hengdeng Liu:** Conceptualization (equal); formal analysis (equal); investigation (equal); methodology (equal); writing – original draft (equal). **Ronghua Yang:** Investigation (equal); methodology (equal). **Shixin Zhao:** Investigation (equal); writing – review and editing (equal). **Fei Zhou:** Formal analysis (equal); project administration (equal). **Yiling Liu:** Investigation (equal). **Ziheng Zhou:** Investigation (equal). **Lei Chen:** Conceptualization (equal); writing – original draft (equal). **Julin Xie:** Conceptualization (lead); funding acquisition (lead); project administration (lead); writing – review and editing (equal).

## CONFLICT OF INTEREST

The authors declare no conflict of interest.

### PEER REVIEW

The peer review history for this article is available at https://publons.com/publon/10.1002/btm2.10467.

## Supporting information


**APPENDIX S1.** Supporting Information

**TABLE S1.** Primer sequence for qPCR
**FIGURE S1.** Fluorescent representative image of BMSCs seeded on the CDRS or micro‐plate at Day 14 (from left to right), Scale bar: 100 μm
**FIGURE S2.** Quantitative analysis of fluorescent intensity for iNOS positive signal in macrophages under different treatment groups
**FIGURE S3.** Quantitative analysis of wound healing at selected times
**FIGURE S4.** Quantitative analysis of the area of the scar tissues
**FIGURE S5.** The quantification of fluorescent intensity for IL‐1β (a), TNF‐α (b), MMP‐9 (c), and IL‐10 (d)

## Data Availability

The main data supporting the findings of this study are available within the paper and its Supporting Information. The associated raw data are available from the corresponding author on reasonable request.
