# Supplementary material for: Collagen scaffolds derived from bovine skin loaded with MSC optimized M1 macrophages remodeling and chronic diabetic wounds healing
Source: Bioeng Transl Med. 2022 Dec 7;8(3):e10467. doi: 10.1002/btm2.10467 (PMC10189465; doi:10.1002/btm2.10467)
Supplement: Supplementary file 1 — APPENDIX S1. Supporting Information [file BTM2-8-e10467-s002.docx]

**Supplementary Materials and Methods**

**2. Materials and Methods**

*2.1. Materials*

Three CDRSs that are authorized in China as potential medical products were used in this study. The collagen extracted from porcine tendons (CPT) (Gunze Limited, Japan) consists of an 0.8-mm-thick sponge layer that was subsequently transformed into atelocollagen. The collagen scaffolds derived from bovine skin (CBS) (ZH-Bio, China) consists of a 0.5-mm-thick heterogeneous decellularized dermal matrix. The collagen from the porcine visceral membrane (CVM) (Guanhao Biotech, China) consists of a layer of collagen of 0.3-mm thickness.

*2.2. Characterization of CDRS*

*2.2.1. Morphological characterization*

The imaging of CDRS with a stereoscope (ZEISS) and by scanning electron microscopy (SEM) (Zeiss Sigma 300, UK) was performed to visualize the surface topography and the construction of the collagen scaffolds.

*2.2.2. Porosity*

The porosity of the sample was measured by using the medium-saturation method. The collagen scaffolds were weighed with a balance (W1). The scaffolds were soaked in anhydrous ethanol until the sample was adsorbed and saturated. A filter paper was then attached to both the sides for 30 s under a certain pressure, after which the scaffolds were quickly weighed and marked as W2. Three parallel experiments were conducted for each sample, and the average value was calculated as follows:

Porosity (%) = (*W2* - *W1*)/*ρV* × 100%

Where, *ρ* is the density of anhydrous ethanol, 0.79 g/cm^3^; *V* is the volume of the scaffolds to be tested, cm^3^.

*2.2.3. Mechanical property*

The mechanical properties of the samples were investigated using an electromechanical universal testing machine (INSTRON 5982, USA) at a tensile speed of 2 mm/min. Before these measurements, the collagen scaffolds were cut into rectangles of dimension 10.0-mm length × 2.0-mm width × 0.1-mm thickness. The samples were stretched under constant temperature and humidity (25° temperature, 70% relative humidity). The tensile modulus was defined as the slope of liner fit to the strain-stress curve. Valid data were obtained three times for each group of samples, and the average value was calculated.

Tensile modulus (E) = Stress (σ)/ Strain (ε)

*2.2.4. Fourier-transformed infrared (FTIR) characterization*

FTIR (Nicolet iS20, Thermo, USA) spectra of CPT, CBS, and CVM were measured at the wavelength of 400–4000 cm^−1^ (numbers of scan, 32; resolution ratio, 4 cm^-1^).

*2.2.5. Water contact angle measurement*

The hydrophilicity of the collagen scaffolds was assessed through a contact angle meter (Dataphysics OCA20, Germany). In accordance with the specific conditions, the time of liquid drop on the samples was maintained for 0.05–0.3 s. The droplet morphology and the contact angle values were recorded.

*2.2.6. Biodegradability assay*

The dried samples were cut into 15 × 15-mm squares and immersed in 37℃ PBS mixed with 10000 U/mL of the lysozyme solution. The samples were then removed on days 7, 14, and 21, respectively, cleaned with ultrapure water, freeze-dried, and, finally, weighed. The *in vitro* mass retention rate of the material was calculated using the following formula:

Weight remaining rate (*%*) = *Wt/Wo* × 100%

Where, *Wo* is the original mass used for incubation and *Wt* is the degraded mass at the incubation time *t.*

*2.3. Preparation of biologically active dermal substitute (BADS)*

*2.3.1. Cell culture of bone marrow stem cells of mice*

C57BL/6 mouse BMSCs cells were provided by the Institute of Precision Medicine, The First Affiliated Hospital of Sun Yat-sen University, China. The cells were cultured in T-75 flasks (Nunc™ EasYFlask™, 75 cm^2^, Thermo Scientific) in Dulbecco's modified Eagle’s medium (DMEM; Servicebio, Wuhan, China) supplemented with 10% fetal bovine serum (FBS; Biological Industries, Israel) and 1% penicillin/streptomycin (P/S) (Gibco) in a wet atmosphere of 5% CO_2_ at 37°C (Thermo Scientific Forma Steri-Cycle i160 Incubator; Germany). The cell culture media were changed every 2–3 days until the cell confluence reached 80–90%.

*2.3.2. Transplantation of mBMSCs onto CDRS*

For the transplantation, ophthalmic surgical instruments were disinfected. Next, a circular skin substitute of 10-mm diameter was obtained using a skin biopsy apparatus and placed on a 24-well plate, with the papillary dermis turned upward. The prepared collagen scaffolds were incubated overnight in fresh complete DMEM, after which 2 × 10^5^ MSCs (between passages 3 and 5) were transplanted on each collagen scaffold and continued to culture in the complete DMEM. Then, CBS-MSCs, CPT-MSCs, CVM-MSCs, and microplate (MP)-MSCs were established. For the *in vitro* analysis, the scaffolds were cultured for up to 2 weeks, and the culture media were changed every 2 days.

*2.4. Cell viability*

The cytotoxicity of MSCs on the skin substitutes was evaluated by using a cell counting kit-8 (MedChemExpress, USA). Briefly, mouse BMSCs were cultured in DMEM supplemented with 10% FBS and 1% penicillin/streptomycin (P/S). For subculturing, 5 × 10^3^ cells/well of MSCs were transplanted on collagen scaffolds into a 96-well culture plate and incubated for 24, 48, 72, and 96 h. Then, the exhausted media was removed and the CCK8 reagent was added in the ratio of 1:9 with the cultured medium, followed by incubation at 37˚C under a 5% CO_2_ atmosphere for 3 h to allow formazan formation. Finally, the absorbance was recorded at 450 nm using an ELISA microplate reader (Thermo Varioskan™ LUX, Finland).

*2.5. Immunocytochemistry staining*

The collagen scaffolds containing MSCs were maintained in 4% paraformaldehyde for 1 h at room temperature on a shaker. Then, the samples were permeabilized for 30 min with 0.1% Triton X-100 and blocked for 1 h at room temperature with 5% bovine serum albumin (BSA) in PBST. The samples were then incubated with primary antibodies diluted in PBST with 1% BSA overnight at 4°C (ki67, 1:250; iNOS, 1:100; CD68, 1:200). Next, the samples were thrice washed thoroughly with PBST and then incubated for 1 h with AlexaFluor-conjugated secondary antibodies (AF488 goats against rabbit, 1:1000; AF555 goats against rabbit, 1:1000; AF488 goats against rat, 1:1000). Phalloidin presented cytoskeleton and 4', 6-diamino-2-phenylindole dihydrochloride (DAPI) was used as a nuclear counterstain. The cell survival test was performed with the Cell Imaging Kit (LIVE/DEAD™ Cell Imaging Kit, Invitrogen). Next, the samples were washed thoroughly with PBST and mounted using the Image-iT™ FX Signal Enhancer (Invitrogen). Finally, the samples were imaged using a confocal laser scanning microscope (Zeiss LSM880, Jena, Germany).

*2.6. Characterization of Conditioned Media by Protein Array*

To obtain a broad profile of the secreted proteins in the conditional medium (CM), a group of antibodies was used to analyze a specific group of molecules. According to the manufacturer's instructions, the Proteome Profiler Mouse XL Cytokine Array (Cat. ARY028, R&D Systems) was used to screen each group of CM for the secreted 111 soluble mouse proteins. The collection and analysis of the pixel density of the light points on X-ray films were performed using a transmission scanner and image analysis software. The data were expressed as the relative integrated density. The antibodies were tested on CM samples from 3 CDRS-MSCs and microplate-MSCs.

*2.7. Expression pattern in LPS-IFNG-induced macrophages*

RAW 264.7 macrophages were obtained from the Institute of Precision Medicine, The First Affiliated Hospital, Sun Yat-sen University. In order to testify to the inflammatory modulation of collagen scaffold toward MSCs, RAW 264.7 were seeded into a 6-well culture plate at the concentration of 2 × 10^6^ cells/well for 24 h, followed by stimulation with the addition of 500 ng/mL of LPS and 10 ng/mL of IFNG to obtain M1-polarized macrophages. After 24 h, the supernatant containing LPS and IFNG were removed, and the cells were gently washed with DPBS thrice. The macrophages were then co-cultured with BADS or MSCs for 12 h. Next, the macrophages were co-stained with CD68 and iNOS, and the cell morphology was assessed by laser scanning confocal microscopy. Meanwhile, the total RNA of the macrophages was extracted and the expression of the relevant markers was analyzed by real-time qPCR. The macrophages with or without LPS/IFNG served as a positive or negative control, respectively.

*2.8. Co-culture of M1 macrophages and BADS*

BADS and MSCs were plated in the upper chamber of a 6-well Transwell plate for culture (0.4-μm-pore size membrane), and the cell density of MSCs was set to 5 × 10^5^ cells/well. Two days before the cell co-culture, RAW264.7 was seeded at 2 × 10^6^ cells/well concentration into the lower chamber and induced into M1-type macrophages by LPS/IFNG. The culture medium of 2 mL was then added to the upper and lower chambers. After incubation, the supernatant and macrophages of the lower chamber were collected for further experiments. Both the cells and supernatants were collected and stored at -80° until further use.

*2.9. Gene expression analysis*

Trizol was used to extract the total RNA from the prepared macrophages. cDNA was prepared from 1 μg of total RNA using the RT First-Strand cDNA Synthesis Kit (Servicebio). qPCR analysis was performed with the CFX Connect Real-Time PCR Detection System (Bio-rad). The primer sequences used are listed in Table S1. All primers used were manufactured by Servicebio. The relative gene expression of each target gene was analyzed by using the 2^−ΔΔCt^ equation (n = 3). The gene expression levels of β-actin served as an endogenous reference.

*2.10. Transcriptome Sequencing*

After the collection of the macrophages as described earlier, Trizol was used to lyse the cells and extract the total RNA. The cDNA libraries were sequenced on the Illumina sequencing platform by Genedenovo Biotechnology Co., Ltd. (Guangzhou, China). The data presented in this study are representative of 3 independent sequencing experiments. The differentially expressed genes (DEGs) were determined by adj. *p*-value < 0.05 and | log2FC | > 1. Kyoto Encyclopedia of Genes and Genomes (KEGG) analysis and Gene Set Enrichment Analysis (GSEA) were conducted to identify the most important biochemical metabolic pathways and signal transduction pathways involved in the differential genes.

*2.11. Enzyme-linked immunosorbent assay (ELISA) analysis*

The supernatant of the macrophages was obtained after centrifugation at 3000 rpm for 15 min. The wound beds were weighed and 0.9% normal saline was added at 1:9 volume. After mechanical homogenization, the homogenate was centrifuged at 2500 rpm for 10 min in a centrifuge tube. These two types of supernatant were collected and stored at -80°C until further use. The levels of TNF-α, IL-1β, IL-10 (Invitrogen, USA), MMP-9, and TGF-β3 (MultiSciences, HangZhou, China) were measured using the ELISA kits as per the manufacturer’s instructions. According to the standard concentration and OD value of the standard curve, the sample concentration was calculated according to the standard curve equation. The results were presented as the amount (pg or ng) of cytokines per milliliter of supernatant.

*2.12. Western blotting*

Macrophages from the selected samples were harvested in the RIPA buffer (MIK) supplemented with PMSF (MIK) and protease and phosphorylase inhibitors (MedChemExpress). Then, the samples were broken with Ultrasonic Cell Disruptor (Scientz, Ningbo). Next, 15 μg of the proteins were separated by SDS-PAGE and transferred onto the polyvinylidene fluoride (PVDF) membranes. These membranes were then blocked with 5% skim milk powder in Tris-buffered saline Tween-20 (TBST) for 1 h at room temperature and treated with the following primary antibodies diluted in 5% BSA/TBST overnight at 4°C: α-tublin, iNOS, CD206, MMP-9, Col3 (1:1000 dilution), phospho-IKKα/β, IKKα, IKKβ, phospho-IκB, IκB, and phospho-NFκB, NFκB (1:1000 dilution). The membranes were washed with TBST and treated with HRP-conjugated secondary antibodies (Goat anti-rabbit, Abcam, 1:10,000) for 1 h at room temperature. The membranes were washed with TBST, developed with the Immobilon Western Chemiluminescent HRP Substrate (Millipore Corp., Merck, USA), and imaged using an imaging analysis system (FluorChem E, ProteinSimple, USA). Densitometry analysis was performed using the ImageJ software, and the protein expression was normalized to the expression of α-tublin as the loading control.

*2.13. Silicone splint and full-thickness skin defect models in db/db mice*

All experimental procedures received the approval and were conducted in accordance with the guidelines of the Experimental Animal Administration Committee of Sun Yat-sen University (Approval Number for Animals: [2022] 241). Male db/db mice aged 6–8 weeks were purchased from Yaokang Biotechnology Co. Ltd (Guangzhou, China). All mice were kept at moderate temperature and humidity before the induction of the formal experiment. The mice were randomized into 5 groups, as follows: Gauze group, CBS-MSCs group, CVM-MSCs group, CPT-MSCs group, and MSCs group. BADS were cultured *in vitro* for 5 days before transplantation into the back wound of mice.

The experimental mice were anesthetized with 2% pentobarbital intraperitoneal injection. The back of the fur was removed with an electric shaving knife. Next, two full-thickness skin defect wounds were created on either side of the dorsum of each mice with a 8-mm circular biopsy punch. To prevent wound contraction, the silicone splints of the corresponding size were sticked up to the wound surface. On days 0, 3, 7, 10, 14, 21, and 28 post-wounding, the wound healing was recorded with a camera, and the residual wound area and wound closure were measured by using an image analysis software (Image J).

*2.14. Hematoxylin-eosin (H&E) and Masson’s trichrome staining*

The wound site and the surrounding normal skin tissues were excised and fixed in 4% paraformaldehyde for 24 h. After fixation, the samples were embedded in paraffin and sectioned (5-μm thickness). Then, the paraffin sections were dehydrated with a gradient series of xylene and anhydrous ethanol. For histological examination, the sections were stained with H&E (G1003, Servicebio) and Masson dye solution set (G1006, Servicebio). Finally, the images were collected and analyzed by a fully automatic pathological section scanner (KF-PRO-020, Ningbo, China).

*2.15. Picrosirius red staining*

Specimens (n = 3) were dewaxed in 100% xylene, followed by washing in ethanol and tap water twice, and then were immersed in Picrosirius red (G1018, Servicebio) for 8 min at room temperature. After washing in tap water, sections were rapidly dehydrated in ethanol, cleared in xylene, and mounted. Collagen fibers were detected by light and polarized light microscopy (NIKON ECLIPSE E100, Japan). Under polarized light microscopy, collagen I (COL 1) fibers were stained red, whereas collagen III (COL 3) fibers appeared green. Images were analyzed using Image J software as previously described [61]. We selected the colour green or red and calculated the area of each collagen type in one field. Three random fields were selected from each sample for statistical analysis.

*2.16. Multiple fluorescence immunohistochemistry*

The paraffin section was deparaffinized, rehydrated, subjected to antigen retrieval, and blocked, followed by the addition of the first primary antibody and then a corresponding secondary antibody marked with HRP. After incubation at room temperature for 50 min, fluorescently labeled-Tyramide Signal Amplification (TSA) (Servicebio) solution was added. The main principle of the TSA is that fluorescent-labeled tyramine becomes activated under the action of HRP and H_2_O_2_, which then attaches to the protein tyrosine residues around the target. After microwave treatment, the second primary antibody and the corresponding HRP-labeled secondary antibody were added. These steps were repeated successively. In this way, multiple targets could be labeled only by changing different fluorescence markers each time. Double or multiple fluorescent staining was performed using different fluorescent tyramines by repeated immunolabeling.

*2.17. Statistical analyses*

Quantitative results were presented as the mean ±standard deviation (SD). Statistical comparisons among each group were performed by Student’s *t*-test or one-way analysis of variance (ANOVA), followed by Tukey’s multiple comparisons to analyze the selected data pairs. *p* < 0.05 was considered to indicate a statistically significant difference.
