# Supplementary material for: Collagen scaffolds derived from bovine skin loaded with MSC optimized M1 macrophages remodeling and chronic diabetic wounds healing
Source: Bioeng Transl Med. 2022 Dec 7;8(3):e10467. doi: 10.1002/btm2.10467 (PMC10189465; doi:10.1002/btm2.10467)
Supplement: Supplementary file 2 — TABLE S1. Primer sequence for qPCR FIGURE S1. Fluorescent representative image of BMSCs seeded on the CDRS or micro‐plate at Day 14 (from left to right), Scale bar: 100 μm FIGURE S2. Quantitative analysis of fluorescent intensity for iNOS positive signal in macrophages under different treatment groups FIGURE S3. Quantitative analysis of wound healing at selected times FIGURE S4. Quantitative analysis of the area of the scar tissues FIGURE S5. The quantification of fluorescent intensity for IL‐1β (a), TNF‐α (b), MMP‐9 (c), and IL‐10 (d) [file BTM2-8-e10467-s001.docx]

**Supplementary Data**

Table S1. Primer sequence for qPCR.

|  | Gene | Forward primer | Reverse primer | |
| --- | --- | --- | --- | --- |
| Mouse | IL-6 | CCCCAATTTCCAATGCTCTCC | CGCACTAGGTTTGCCGAGTA | |
|  | IL-10 | ATGCTGCCTGCTCTTACTGACTG | CCCAAGTAACCCTTAAAGTCCTGC |  |
|  | β-actin | GTGACGTTGACATCCGTAAAGA | GTAACAGTCCGCCTAGAAGCAC | |
|  | IL-1β | GTATGACTCTACCCACGGCAAGT | TTCCCGTTGATGACCAGCTT | |
|  | Arg-1 | ATCAACACTCCCCTGACAACCA | TTCCATCACCTTGCCAATCC | |


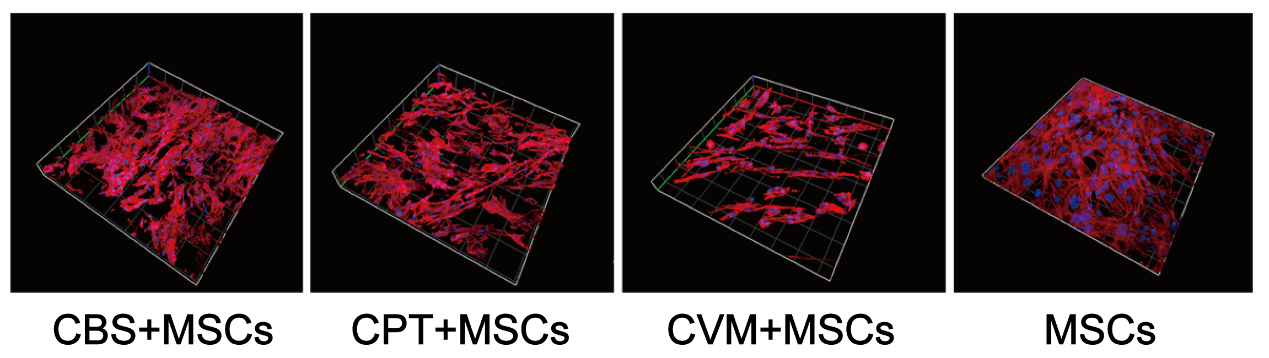


**Figure S1.** Fluorescent representative image of BMSCs seeded on the CDRS or micro-plate at day 14 (from left to right), Scale bar: 100μm**.**


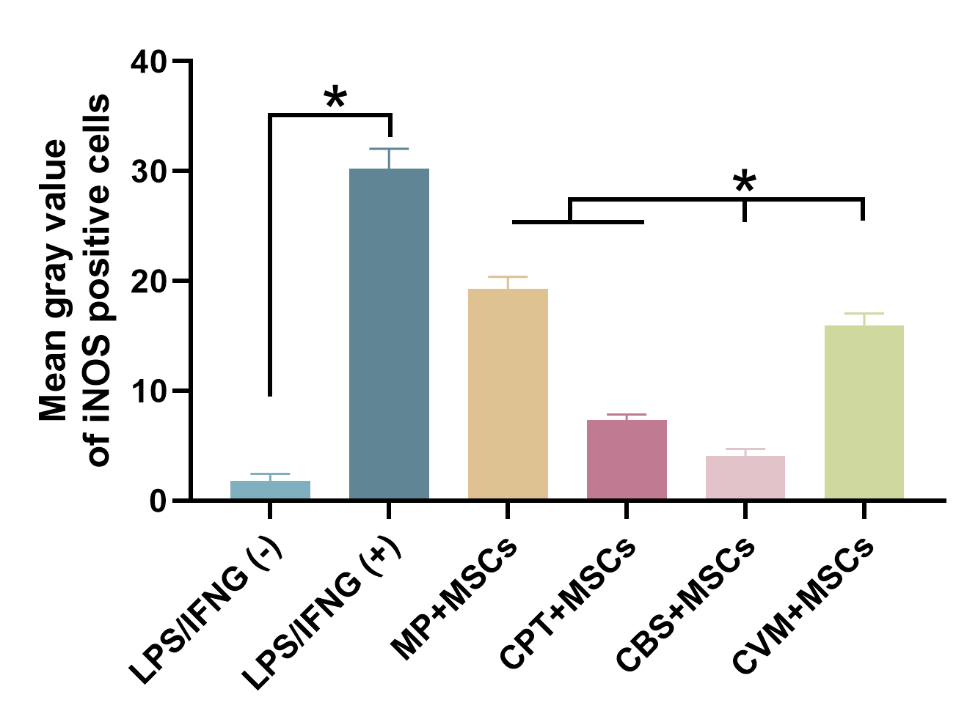


**Figure S2.** Quantitative analysis of fluorescent intensity for iNOS positive signal in macrophages under different treatment group.


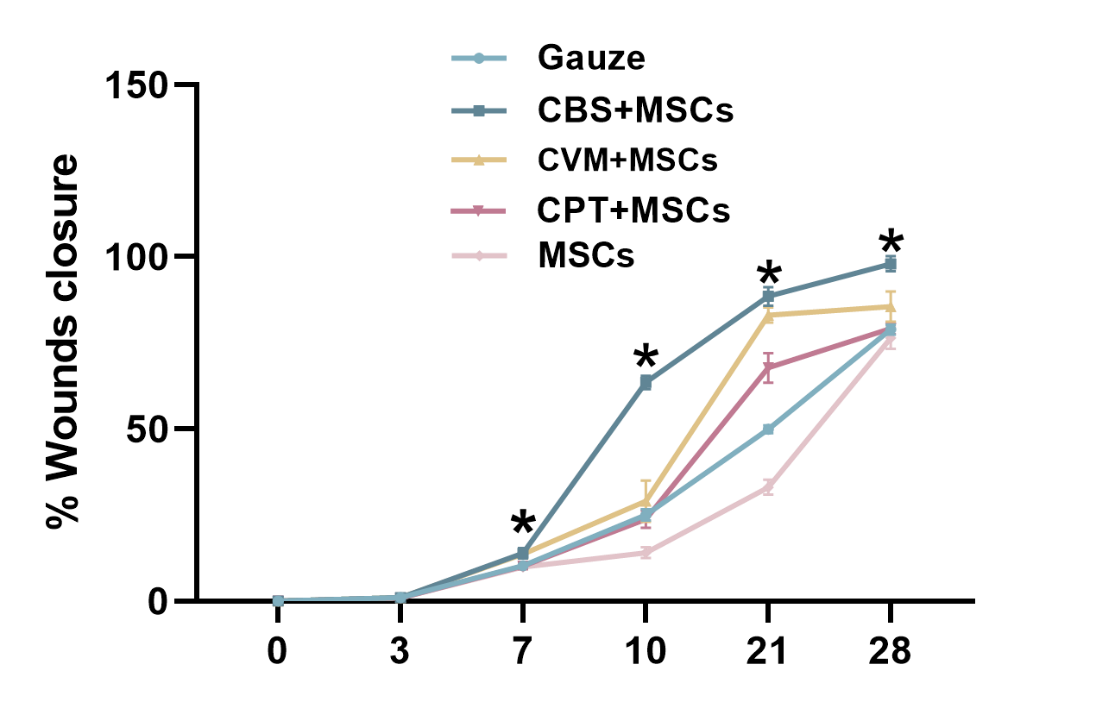


**Figure S3.** Quantitative analysis of wound healing at selected times.


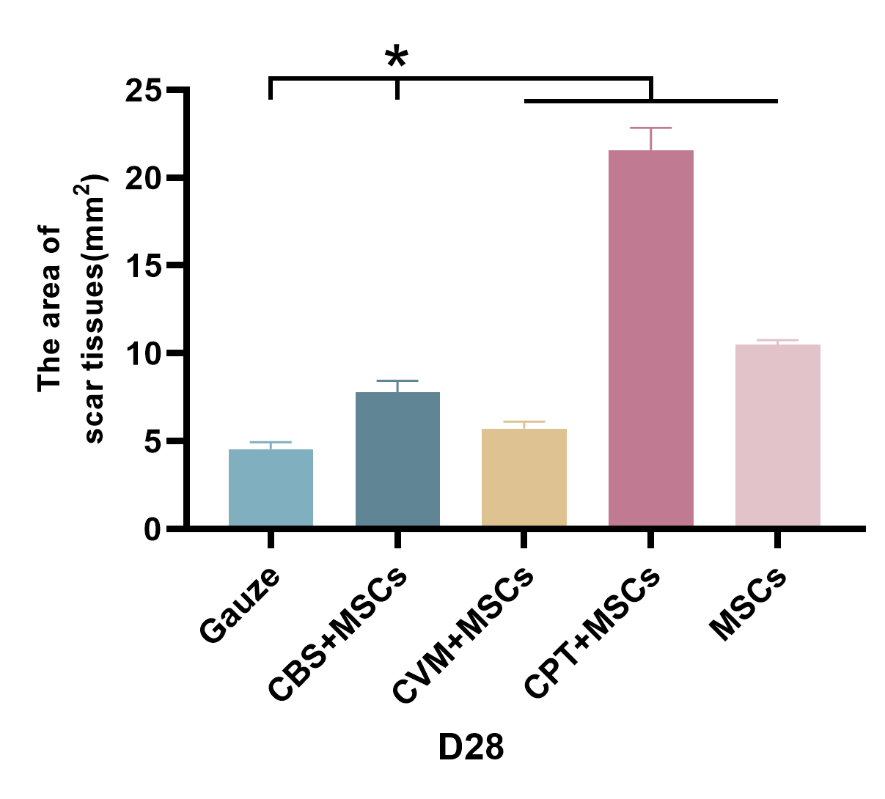


**Figure S4.** Quantitative analysis of the area of the scar tissues.


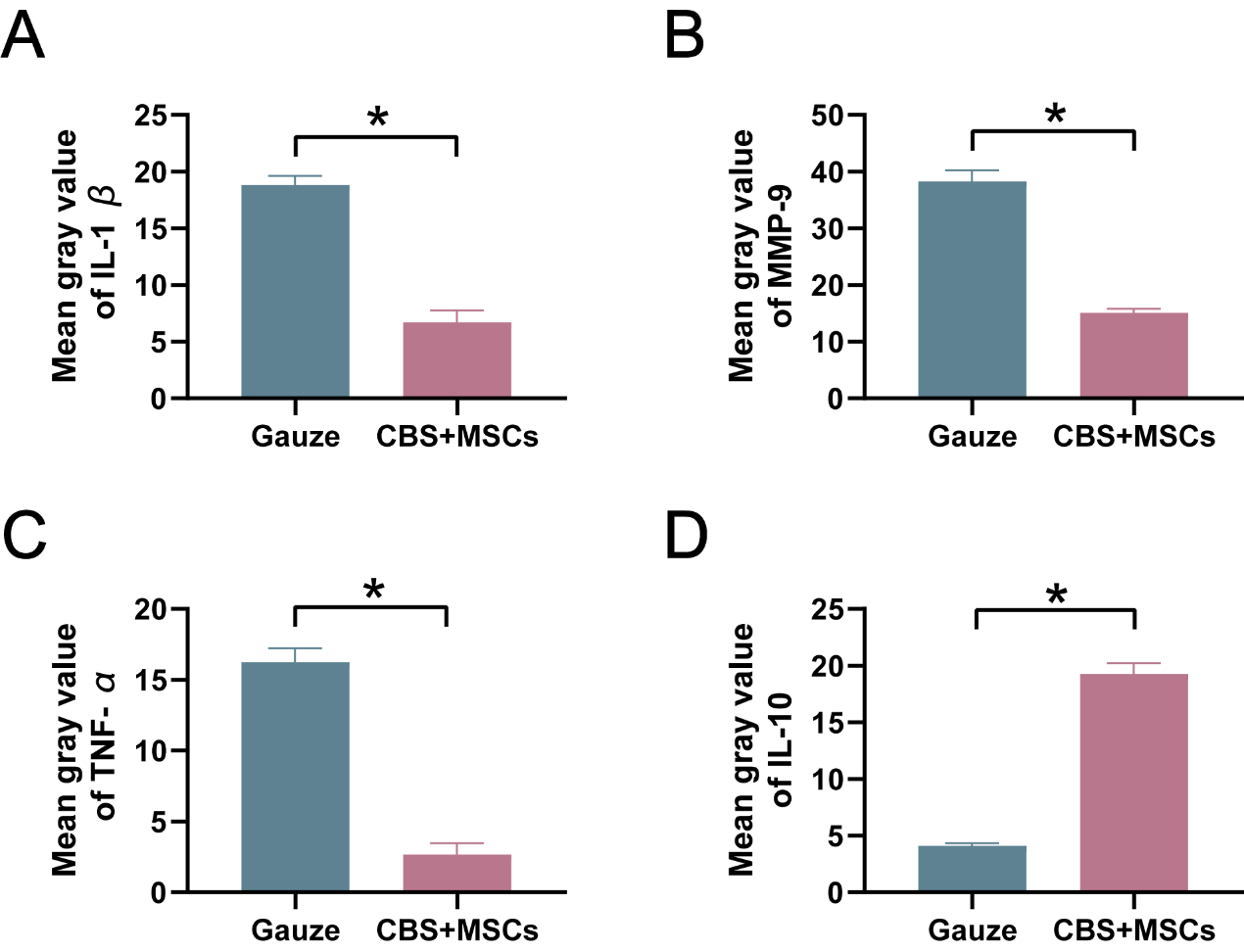


**Figure S5.** The quantification of fluorescent intensity for IL-1β (A), TNF-α(B), MMP-9 (C) and IL-10(D).
